# Systematic Review on the Association Between Sleep and the Risk of Alzheimer’s Disease: An Evolutionary Perspective

**DOI:** 10.1111/eva.70223

**Published:** 2026-03-23

**Authors:** S. E. Spirig, S. Frei, A. V. Jaeggi, N. Bender

**Affiliations:** ^1^ Institute of Evolutionary Medicine University of Zurich Zurich Switzerland

## Abstract

Alzheimer's disease (AD) is an important, non‐curable disease today. Several possible risk factors have been discussed, among others sleep. Evolutionary hypotheses were proposed to explain sleep variation and AD risk, such as a potential advantage of short or interrupted sleep in ancient insecure environments, the evolution of increased plasticity of the human brain, or antagonistic pleiotropy with increased AD risk. The aim of this systematic review was to investigate in which way sleep is associated with AD risk, in the light of evolutionary hypotheses. Following PRISMA guidelines, the databases PubMed and Embase were searched for longitudinal observational studies on the association between sleep and incident AD in cognitively healthy people at baseline. Potential confounders were assessed (e.g., age, sex, country, etc.). Search results were deduplicated and assessed for inclusion or exclusion by two independent reviewers. We summarized the results in tables and performed meta‐analyses in risk factor subgroups where appropriate. Out of 5800 studies, 39 were suitable for this review and 35 were meta‐analyzed. Long sleep duration showed a positive association with AD (HR = 1.35, 95% CI = 1.12–1.63) that was not significant after accounting for heterogeneity using prediction intervals (95% CI = 0.74–2.49). Short sleep duration showed a weak, non‐significant association with AD risk (HR = 1.07, 95% CI = 0.98–1.18). Several measures of sleep quality (hypnotics use, daytime sleepiness, bad overall sleep quality, and early bedtime) showed an increased risk for AD, while others (e.g., late bedtime) showed a protective effect. This systematic review showed possible associations between sleep characteristics and the risk for AD. However, several results showed heterogeneity that we could not explain with the information given in the publications. More work is therefore needed to assess the risk factors for AD connected to sleep.

## 1 Introduction

Alzheimer's disease (AD) is a leading health problem of our time in Westernized societies. More than 100 years ago the first case of AD was described (Yang et al. 2016). In 2020, around 50 million people suffered from dementia and, with 60%–70% of patients, AD contributes the largest number of cases (Guerchet et al. 2020). There is an enormous social and economic burden linked to AD, with worldwide costs of 1.3 trillion US dollars in 2021 alone (WHO 2024). Despite over a century of intensive research, therapeutic breakthroughs are still missing. Each year, more than 10 million new dementia cases worldwide are registered, and the count is expected to increase up to 152 million people suffering from dementia by 2050 (Guerchet et al. 2020).

Research found multiple risk factors for AD, for example medical conditions like diabetes type II, hypertension, orthostatic hypotension, or depression (Yu et al. 2020), lifestyle factors such as low cognitive activity, high stress, smoking (Yu et al. 2020), unhealthy dietary patterns (Booth et al. 2025), or poor oral health (Pruntel et al. 2024), as well as environmental factors such as air pollution or pesticides (Jones et al. 2025). Genetic factors are discussed to affect the susceptibility to AD as well, notably the ε4 allele of Apolipoprotein E (APOE E4). Studies indicate carriers of this allele have an up to 10 times increased risk for AD (Bufill et al. 2013). On the other hand, there are hints that heterozygous carriers of APOE E4 may profit from better cognitive performance at middle age or higher fertility (Trumble et al. 2023) depending on environmental factors, such as, for instance, in non‐industrialized societies with higher parasite burden (Fox 2018; Trumble et al. 2017), hinting toward intriguing gene–environmental interactions.

Today's research on risk factors for AD points to sleep as an important factor in the pathophysiology of neurodegenerative diseases, and particularly of AD (Nesse et al. 2017; Guillot et al. 2025; Lucey 2020). Studies highlight the importance of the restorative function of the glymphatic system (Astara et al. 2024) removing Amyloid beta (Aβ) plaques from the brain twice as actively during sleep than during the day (Xie et al. 2013). On the other hand, the sleep related neurotransmitter melatonin shows an inhibitory effect on Aβ polymerization (Poeggeler et al. 2001) and its anti‐inflammatory characteristics (Bocheva et al. 2024). There are hints that only one night of sleep deprivation increases the level of Aβ in the brain up to 30% (Lucey et al. 2018). Previous studies investigating sleep duration and its potential role in the pathophysiology of AD found a U‐/or J‐shape association, representing an increased risk for AD associated with changes of sleep duration in both directions (Li et al. 2024; Fan et al. 2019; Wang et al. 2024). Furthermore, sleep quality characteristics such as insomnia, sleep timing, or sleep efficiency showed associations with the risk for AD (Ferini‐Strambi et al. 2024).

Considering the increasing global impact of AD and its limited therapeutic options, the present study aims to investigate another aspect of the disease, namely etiology. What do we know about risk factors, and how can we explain human vulnerability to AD from an evolutionary point of view? The change of perspective shall help us to detect hints that may have been missed along the way and open new opportunities for prevention and therapy. The sleep of modern humans has changed over evolutionary times. With an average sleep duration of 7–8 h, modern humans have shifted to a unique short sleep pattern compared to longer sleep duration (9–10 h) among other primates (Nesse et al. 2017), and modern humans show the highest proportion of rapid‐eye‐movement (REM) sleep (Nunn et al. 2016; Samson and Nunn 2015). Interestingly, other animals such as cats, dogs, and primates show similar changes in cognitive capacities at old age as humans, such as a decrease in learning capacity or memory (Gołaszewska et al. 2019). Aging primates such as macaques also show similar tau and amyloid cortical pathologies as aging humans (Paspalas et al. 2018). However, it is important to discern physiological changes in senile involution and neurodegeneration that lead to dementia. In fact, correlations between behavioral changes and neuropathological lesions seem to be different in other primates than in humans. More precisely, distinct clinical and pathological stages, and therefore distinct aging models, may be present in different primate species, including humans (Toledano et al. 2012).

Today's short sleep duration was hypothesized as a tradeoff between benefits of daily activities, for example, learning and socializing, and the cognitive benefits from sleep, for example, better attention or decision‐making abilities (Fox 2018; Nunn et al. 2016). Furthermore, as the high prevalence of AD is characteristic for modern humans (Bufill et al. 2013; Finch and Austad 2015), the question arose if selection for short sleep duration may explain humans' vulnerability to AD. In this respect, AD is seen as a side product of the natural selection for shorter sleep. This negative side product would have been only weakly selected against, if at all, as the first symptoms of AD normally start after the reproductive phase (Nesse et al. 2017).

A further evolutionary hypothesis on the association between sleep and AD stresses the aspect of mismatch in modern environments, as the mentioned missing association between the risk allele APOE E4 and AD in more traditional societies suggests. According to the mismatch hypothesis, triggers from the modern Western lifestyle are necessary to provoke the positive association between APOE E4 and AD (Fox 2018). As non‐Western societies not only miss the risk association but also seem to profit from increased cognitive abilities at mid‐age, as mentioned above, antagonistic pleiotropy was proposed to explain the maintenance of this risk allele in the gene pool (Diederich et al. 2025). Antagonistic pleiotropy means that certain genetic variants may confer beneficial effects early in life while causing detrimental effects later in life.

The aim of this study is therefore to perform a systematic review of today's knowledge about the potential role of sleep characteristics as risk factors for AD, and to integrate and discuss the results from an evolutionary point of view. This approach may lead to novel insights into the pathophysiology and prevention of AD.

## 2 Methods

This systematic review was performed according to the PRISMA guidelines (Page et al. 2021), and followed a predefined study protocol. The protocol was pre‐registered on the platform PROSPERO (CRD42024497332). We included longitudinal observational studies that investigated the association between sleep patterns or diseases associated with sleep (e.g., sleep apnea) and the risk for AD in human adults from the general population and not already burdened with AD or any other cognitive impairment at baseline. Studies were only considered if AD was either diagnosed by an AD specialist, a specialized consortium, or according to the relevant ICD codes, during life or postmortem. Studies were not considered if the outcome relied solely on biomarkers (e.g., Amyloid beta (Aβ)), imaging, or genetic risk alleles. Only papers published in English were considered as well as only freely accessible work. There was no restriction on the publication date. Animal testing was an exclusion criterion, as well as AD drug testing. Studies on drugs were only considered if the drug was used as treatment of a sleep affecting disease (e.g., hypnotics, obstructive sleep apnea (OSA) treatment) and could therefore be used as a proxy for sleep disturbances.

We searched the databases Embase and PubMed, using predefined search terms; the search date was 17.01.2024, and the last update was 15.10.2025. The search terms included terms related to sleep characteristics, AD, and study design (longitudinal, observational studies). We excluded conference abstracts, letters to the editor, and other incomplete study reports. Where possible we used medical subject headings (MESH) terms. The full search strategy can be found in Appendix S1.

**FIGURE 1:**
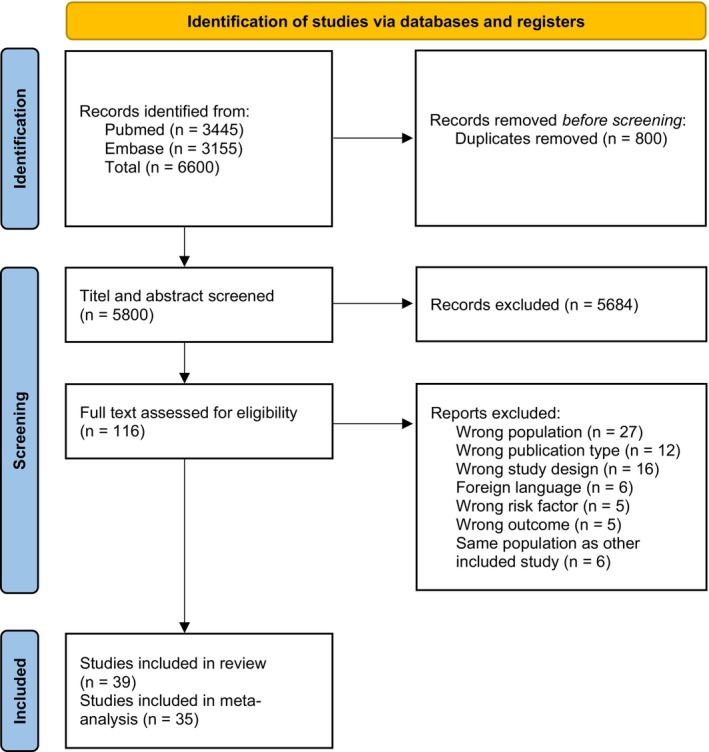
PRISMA 2020 flow diagram showing included and excluded studies.

The resulting studies were uploaded into the online systematic review software Rayyan. Up to three independent reviewers screened the titles and abstracts for inclusion or exclusion using predefined criteria. Conflicting decisions were solved by discussion. The remaining studies were further screened in full text for inclusion and exclusion, giving a reason for exclusion (see Figure 1). We extracted bibliographic data, data on the population included, study type and duration, risk factor analyzed, and outcome assessment, as well as measures of association and potential confounders on a predefined extraction sheet and presented the results in a table (Table 1). The quality of the studies included was assessed using the Newcastle‐Ottawa Scale (NOS) (Wells et al. 2000). The results of the quality assessment were added to Table 1.

**TABLE 1:** Description and quality of included studies.

Study Nr.	References	Population	Study design	Country	*N* per group	Risk factor (Intervention)	Duration of study	Outcome assessment	Nos
1	Baek et al. (2021)	NHIS (National Health Insurance Service) > 40 years diagnosed with AD/VaD between 2007 and 2014	Cohort	Korea	Insomnia: 2,796,871 No insomnia: 5,593,742	Insomnia	8 years	ICD 10 Codes related to Insomnia and AD and VaD (G47, F00, G30)	7
2	Baril et al. (2021)	Framingham Heart Study Offspring cohort mean age: 67.5 ± 4.9 years 51.6% men	Cohort	USA	291	Sleep quantity and quality	Average: 13.4 ± 5.4 years, up to 22.5 years	Criteria of the National Institute of Neurologic and communicative Disorders and Stroke, as well as the AD and Related Disorders Association	7
3	Benedict et al. (2015)	Uppsala Longitudinal Study of Adult Men (ULSAM) men at ages 50: *n* = 1574 men at ages 70: *n* = 1029	cohort	Sweden	Ages 50: with sleep disturbances 342, without 1232 ages 70: with sleep disturbances 236, without 793	Self‐reported sleep disturbances	40 years (reviewing patient history between ages 50 and 90 years) 35.380 years at risk	Criteria of the National Institute of Neurological and Communicative Diseases and Stroke and the AD and Related Disorders Association	7
4	Benito‐León et al. (2014)	NEDICES Study aged ≥ 65 years	Cohort	Spain	3857	Daily sleep duration 3 categories: ≤ 5 h (short sleepers) 6–8 h (reference) ≥ 9 h (long sleepers)	12.5 years	Dementia mortality risk in short or long sleepers relative to the reference group Dementia condition reported on the death certificate excluded all participants (*n* = 53) in whom a diagnosis of non‐Alzheimer disease dementia was listed on the death certificate	6
5	Burke et al. (2016)	National Alzheimer's Coordinating Center (uniform data set UDS)	Retrospective cohort	USA	Total 11,453 Sleep disturbance present: 1120 (10.6%) Sleep disturbance absent: 9456 (89.4%)	sleep disturbance =nighttime behaviors (awakening during the night, rising too early in the morning, excessive naps during the day) Issues with sleep were measured utilizing NPI‐Q	286 days until the first occasion that AD diagnosis occurred, the maximum was 3229 days (Mean: 1469.37 d, Median: 1350.5 d)	Sporadic late‐onset AD (probable AD) UDS using criteria set forth by the National Institute of Neurological and Communicative Disorders and Stroke and the AD and Related Disorders Association	7
6	Cavaillès et al. (2022)	Of three different french cities	Cohort	France	9294	Sleep Quality (SQ) Sleepiness during The Day (EDS) Difficulty initiating sleep (DIS) Difficulty maintaining sleep (DMS) Early morning awakening (EMA) Hypnotics use	12 years	DSM‐IV criteria and validated by neurologists	7
7	Cavaillès et al. (2024)	Three city studies	Cohort	France	6171	Excessive daytime sleepiness	12 years	DSM‐IV criteria and validated by neurologists	6
8	Chung et al. (2025)	NHIS with sleep apnea and control group > 50	Cohort	Korea	Sleep apnea: 30,111 (30,382) Control: 121,528 (119,043)	Sleep apnea G47.30, G47.31, G47.32, G47.38	17 years	KCD‐7, ICD 10 and death records	8
9	Gang and Chen (2025)	UK Biobank	cohort	China	OSA: 9228 control: 435,795	OSA confirmed through ICD‐10 codes	12.6 years	Determined through algorithmic methods incorporating mortality register date, hospital inpatient records, and self‐reported data, dementia definitions were based on specific ICD‐10 codes	8
10	Gao et al. (2025)	Rush Memory and Aging Project (MAP)	Cohort	USA	936	Daytime napping measured by actigraphy	17 years	Criteria of the National Institute of Neurological and Communicative Disorders and Stroke and the Alzheimer's Disease and Related Disorders Association	8
11	Gribsholt et al. (2025)	The Danish National Patient Registry (DNPR)	Cohort	Denmark	OSA: *n* = 62,928 Control: *n* = 628,444	OSA confirmed through ICD‐10 codes	Median follow‐ up: 7.2 y	Defined on the basis of inpatient and outpatient diagnostic codes from the DNPR and the DCPRR (Danish Central Psychiatric Research Registry)	7
12	Hahn et al. (2014)	Kung‐sholmen Project aged ≥ 75y	cohort	Sweden	214	Change in sleep pattern: Self‐reported reduced duration and/or depth, measured by CPRS Rating Scale 1. Dichotomous variable: Sleep reduction > 2 h [CPRS score ≥ 1] versus sleep reduction < 2 h [CPRS score < 1] 2. Dose response analyses, 3 groups none [CPRS 0 or 0.5] mild [CPRS 1] moderate/severe [CPRS score ≥ 1.5] 3. Dose response analyses, continuous variable CPRS score: 0 = absence of symptoms 1 = occasional symptoms 2 = continuous symptoms 3 = “extreme” change from the person's normal behavior additional answer choices (i.e., 0.5, 1.5, 2.5)	9 years	Physician diagnosed (AD criteria using the Hachinski et al. scale or according to the National Institute of Neurological and Communicative Disorders and Stroke—Alzheimer's Disease and Related Disorders Association)	7
13	Huang et al. (2025)	UK Biobank ADNI	Cohort	UK USA Canada	321,905 UK Biobank 1598 ADNI	Sleep quality	13 years UK Biobank mean: 12.3 years ADNI mean: 3.9 years	UK Biobank: ICD‐9: 331.0 ICD‐10: F00, G30 ADNI: National Institute of neurological disorders and stroke	7
14	Kim et al. (2023)	Korean National Health Insurance Service‐Elderly Cohort Aged ≥ 60y	Retrospective cohort study	Korea	RLS: 2501 Control: 9977	Restless leg syndrome (RLS)	12 years	ICD‐10: F00 or G30	8
15	Kummer et al. (2019)	Claims data from a 5% random sample of Medicare beneficiaries Aged ≥ 66y	Retrospective cohort study	USA	Total 1,035,536 ad: 81,974 (OSA 3.1%) No AD: 953,562 (OSA 2.9%)	Cardiovascular risk factors, one of it obstructive sleep apnea (OSA)	5.2 years	ICD‐9‐CM: 331.0	7
16	Larsson and Wolk (2018)	National Research Infrastructure SIMPLER from different swedish counties aged ≥ 65y	Population‐based cohort study	Sweden	28,775	Sleep duration To evaluate whether reverse causation bias may have influenced the results for sleep duration and AD risk, the analysis was repeated after exclusion of AD cases diagnosed within the first five or 10 years of follow‐up.	12.6 years	Swedish National Patient Register	7
17	Lee et al. (2019)	Health Check‐up of the NHIS between 2002 and 2003 (2015)	Cohort	Korea	Sleep disordered breathing: 727 control: 3635	Sleep disordered breathing, CPAP or bilevel positive Airway pressure treatment	13 years	ICD‐10 G30	7
18	Li et al. (2023)	Rush Memory and Aging Project (MAP)	Cohort	USA	1203 in baseline daytime sleeping to ad 1065 in researching changes in daytime sleepiness in dementia	Daytime nap duration	23 years	Criteria recommended by the National Institute of Neurological and Communicative Disorders and Stroke and the Alzheimer's Disease and Related Disorders Association (NINCDS/ADRDA)	7
19	Lin et al. (2015)	LHID	Cohort	Taiwan	Sleep related movement disorders (SRMD): 604 control group: 2416	Sleep related movement disorders	10 years	International Classification of Diseases, Ninth Revision, Clinical Modification (ICD‐9‐CM): codes 290.1 and 331.0	8
20	Liu et al. (2022)	SYS‐AD & MIND	Population based cohort	China	incident dementia *n* = 1982	Sleep duration Time in Bed (TIB) Bed Time Pittsburgh sleep quality Index Epworth sleepiness Scale	4 years	DSM‐IV criteria and NIA‐AA criteria (National Institute on Aging‐Alzheimer's Association (NIA‐AA))	6
21	Luojus et al. (2016)	Kuopio Ischemic Heart Disease Study (KIHD) only men, mean age 53 ± 5.2y	Prospective cohort study	Finland	2386	Self‐reported sleep disturbances: difficulty falling asleep or maintaining sleep, sleep duration and daytime tiredness	21.9 ± 7.9 years	Physician diagnosed and certificate assessed in Special Reimbursement Register (SRR) maintained by the Social Insurance Institution (SII) of Finland (diagnostic criteria of DSM‐IV and NINCDS‐ADRDA)	7
22	(Lutsey et al. 2019)	Atherosclerosis Risk in Communities (ARIC) Study participants as part of the Sleep Heart Health Study (SHHS) average 62.7 ± 5.5 years old 52.6% female	Prospective cohort study	USA	1667	Obstructive sleep apnea (OSA) (in‐home polysomnography) short and long sleep duration (SHHS Sleep Habits Questionnaire)	15 years	Criteria from the National Institute on Aging‐Alzheimer's Association (NIA‐AA) workgroups “based on the presence of the cognitive syndrome that is not of abrupt onset and includes memory impairment and the absence of features of other specific diagnoses sufficient to cause the cognitive impairment”	7
23	Lysen et al. (2018)	The Rotterdam Study mean age 72 years 58% women	Prospective population‐based cohort study	Netherlands	4835	Subjective sleep quality (home interview, Pittsburgh Sleep Quality Index PSQI)	13 years (mean 8.5 years)	Physician diagnosed criteria based on NINCDS–ADRDA (National Institute of Neurological and Communicative Disorders and Stroke and the Alzheimer's Disease and Related Disorders Association)	7
24	Lysen et al. (2020)	The Rotterdam Study mean age 66 ± 8 years 53% women	Prospective population‐based cohort study	Netherlands	1322	objective estimates of sleep and 24‐h activity rhythm measured by actigraphy	11.2 years	physician diagnosed criteria based on NINCDS–ADRDA (National Institute of Neurological and Communicative Disorders and Stroke and the Alzheimer's Disease and Related Disorders Association)	8
25	Ohara et al. (2018)	The Hisayama Study aged 60 and older	Prospective cohort study	Japan	1517 (667 men, 850 women)	Daily sleep duration (self‐administered questionnaire)	10 years (mean 8.8 years)	Physician diagnosed (ICD‐10) Criteria of the NINCDS‐ADRDA used 180 underwent an autopsy	7
26	Schneider et al. (2022)	NIH‐AARP aged 50–71	Cohort	USA	N1 = 332,674 napping N2 = 333,373 sleep duration	Sleep duration (long)	15 years	Death due to AD determined by ICD‐9 and ICD‐10 codes	7
27	Selbaek‐Tungevåg et al. (2023)	Norwegian Trøndelag Health Study (HUNT)	Retrospective cohort study	Norway	7082	Probable insomnia disorder (PID) according to DSM‐5 criteria Insomnia symptoms – difficulties initiating sleep (DIS)—difficulties maintaining sleep (DMS)—early morning awakenings (EMA)	11 years	Physician diagnosed, using DSM‐5 criteria	7
28	Sun et al. (2025)	UK Biobank mean age 56.5 (SD 8.1) years at baseline	Prospective cohort study design	United Kingdom	429,761	Sleep duration (hours) 4 groups: 0–6 h 6–9 h 9–11 h ≥ 11 h	Mean 11.2 (SD 1.6) years	ICD‐10: F00, G30 ICD‐9: 290–1	7
29	Tsai et al. (2020)	NHIRD > 40 Years with OSA between 1997 and 2012	cohort	Taiwan	3978 OSA 15,912 non OSA	OSA	16 years	AD ICD‐9 Code 331.0	8
30	Virta et al. (2013)	Twin cohort	cohort	Finland	2336	Sleep duration (hours) 4 groups: 0–6 h 6–9 h 9–11 h ≥ 11 h	Follow up‐Time mean 22.5 (range 15.8–25.7) SD 2.2	MMS, Drug Use (through Social Insurance Institution of Finland) and individuals who had been prescribed cholinesterase inhibitors (donepezil, rivastigmine or galantamine) or memantine prior to this were considered to have AD. Because the only indication for these pharmaceuticals in Finland is AD, and off‐label use of medications is not reimbursed.	7
31	Wang et al. (2025)	CPRD	Open cohort	UK	OSAS positive: 193,600 OSAS negative (control): 536,701	OSAS	22 years	The secondary outcomes were the two main subtypes of dementia, Alzheimer's disease and vascular dementia Diagnoses were recorded as read codes/SNOMED‐CT in CPRD‐Gold and CPRD Aurum	8
32	Westwood et al. (2017)	The Framingham Heart Study (FHS) mean age: 72 ± 6 y 57% women	Prospective cohort study	USA	2457	Sleep duration (self‐reported) < 6 h (short) 6–9 h (reference) > 9 h (long)	10 years	Based on NINCDS‐ADRDA Criteria for definite, probable, or possible AD	7
33	Wong et al. (2023)	Million Women Study	Cohort	UK	830,716 Women	sleep duration (self‐reported) < 7 h (short) 7–8 h (normal) ref. > 8 h (long) and daytime napping	Mean follow up 16.6 years	ICD‐10 Codes F00‐F03 & G30 electronic linked in the NHS	7
34	Xiong et al. (2024)	English Longitudinal Study of Aging	Observational Study	Sweden	8375	sleep disturbances assessed through the following questions: difficulty falling asleep, waking up several times during the night, and waking up with tiredness	10 years	Physician diagnosis of AD during the follow‐up until wave 9 (2018/2019)	6
35	Yaffe et al. (2015)	Department of Veterans Affairs (VA) National Patient Care Database aged ≥ 55 years only men (veterans)	Retrospective cohort study	USA	179,738	Sleep disturbance (ICD‐9) any sleep disturbance (780.5) apnea (780.51, 780.53, 780.57) insomnia (780.52)	8 years	International Classification of Disease, Ninth Revision Code 331.0	7
36	Zhao et al. (2025)	UK Biobank	Cohort	UK	88,592	Sleep duration	6.62 years	ICD‐9 and ICD‐10 codes from medical history, hospital records, and mortality data developed by the UK Biobank	7
37	Allwright et al. (2023)	UK Biobank aged 60–70	Cohort	UK	156,209	Sleepiness/insomnia	8.2 years	ICD10 codes: G30.0, G30.1, G30.8, G30.9	7
38	Atayde et al. (2022)	National Alzheimer's Disease Coordinating Center (NACC)	Cohort	USA	No NTB: 330; NTB: 74	Nighttime behavior (NTB) as unusual sleep patterns	3.4/3.5 years plus max 2 years	Post mortem: Autopsy‐confirmed primary neuropathologic diagnosis of AD based on the NACC classification system as described in the NACC NP Data Element Dictionary and Diagnosis Coding Guidebook (Version 9.1, September 2008)	6
39	Dunietz et al. (2021)	Medicare 5% fee‐for‐service claims data (Medicare Beneficiary Summary File in 2011, 2012, and 2013) aged ≤ 65	Retrospective cohort study	USA	Total: 53,321 with OSA (obstructive sleep apnea) diagnosed with ICD‐9 codes prior to 2011 PAP treated: 41,466* * treatment adherent: 30,717 * non‐adherent: 10,749 PAP untreated: 11,855 Treatment = presence of one or more HCPCS (healthcare Common Procedure Coding System) claims code Adherent = two more HCPCS claims, separted by at least 1 month from each other	PAP treatment and adherence of OSA	3 years	ICD‐9 codes	6

Studies reporting results in a suited format were meta‐analyzed (see below). Some papers did not meet the criteria to be included in the meta‐analysis. Papers 37 (Allwright et al. 2023) and 38 (Atayde et al. 2022) did not report results in a way suited for meta‐analysis. Paper 39 (Dunietz et al. 2021) did not report the risk for AD due to sleep disturbances, but how much non‐disturbed sleep or the use of CPAP was lowering the risk for AD. If the results of a study were reported in more than one publication, we chose the publication with the most complete or relevant results for our research question.

### 2.1 Statistical Analysis

35 studies reported results suited for meta‐analysis. Most studies reported their results as hazard ratios (HR), a few studies reported their results as risk ratios (RR) or odds ratios (OR). As the risk for AD in the age group 65–84 is estimated to be at ca 7.7% (NIH National Institute on Aging (NIA) 2024), we accepted an approximation of HR, RR, and OR (VanderWeele 2017). The meta‐analyses were performed separately by risk factors (long sleep duration, short sleep duration, sleep quality, and OSA) and using random effects models. Long sleep was commonly defined as > 9 h of sleep, while short sleep duration was mostly defined as < 6 h of sleep. The category of sleep quality encompasses several sleep‐related disturbances, such as insomnia, frequent awakening during the night, early awakening, restless leg syndrome (RLS), etc., and might reflect diverse phenotypes with potentially distinct mechanisms. Where possible, the results of this category were therefore analyzed separately by specific type of sleep disturbance.

Heterogeneity was analyzed using the *I* ^2^ statistics (Higgins and Thompson 2002). Additionally, we calculated prediction intervals in heterogeneous meta‐analyses with more than 8 studies. In fact, studies in a meta‐analysis may show heterogeneous results because of differences in study populations, risk factors, or outcome measurement methods, follow‐up length, or publication bias. As the *I* ^2^ statistics show the percentage of the total variability due to true differences between the included studies but tell little about clinical interpretation, prediction intervals are used to show which range of true effects can be expected in future settings (IntHout et al. 2016). Furthermore, in the case of heterogeneity, the potential reasons were explored using meta‐regressions, and we explored potential small study bias and publication bias using funnel plots and the Egger test (Egger et al. 1997). Meta‐analyses and meta‐regressions were performed with the statistical software STATA (StataCorp. 2015. Stata Statistical Software: Release 14. College Station, TX: StataCorp LP).

## 3 Results

After deduplication we found 5800 studies with our search strategy in Pubmed and EMBASE. Of the 5800 studies initially found, 116 were assessed in full text, and 39 were deemed suitable for this paper and 35 were suitable for meta‐analysis. For all results of inclusion and exclusion of studies, see Figure 1.

We presented the 39 studies with relevant outcomes in a descriptive table (Table 1) where we reported the type of study, the country of the study, the number, age, and sex of included participants, the risk factor assessed and the method of assessment, the method to diagnose the outcome AD, and study quality. Most studies were cohorts carried out in the US, Europe, or Asia and included several thousand participants. Mostly the sleep characteristics were assessed by questionnaires, and most of the AD diagnoses were done in living elderly using standardized assessment tools and including several experts in assessment panels. Most studies corrected their results for several potential confounders such as age, sex, co‐morbidities, and socioeconomic status. The results of the quality analysis with NOS showed that all studies are of good quality (6–8 points out of max 9 points) (Table 1).

For the meta‐analyses, the studies were divided into categories of long sleep, which consisted of 13 papers; short sleep, which consisted of 12 papers; OSA, which consisted of 9 papers; and sleep disturbance, which consisted of 19 studies and was divided into 15 different subgroups (see details below). In the descriptive table, we included one study twice (Lysen et al. 2020, 2018) of which the earlier version reported a condensed outcome measure, while the later version reported more details, for example, the possible influence of bedtime or the influence of disturbances of the 24‐h rhythm, etc., on AD risk. Another paper (Westwood et al. 2017) showed non‐significant results concerning long or short sleep duration but significant results on sleep patterns, specifically the change from short to long sleep duration as an increased risk factor for AD.

### 3.1 Results of Meta‐Analyses

**FIGURE 2:**
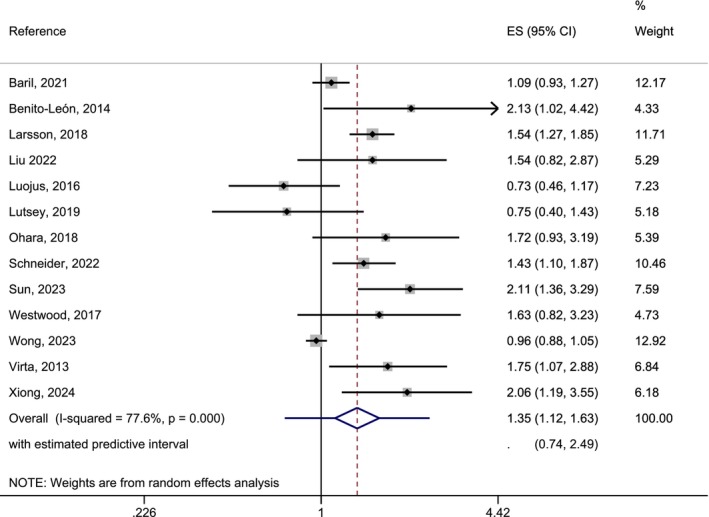
Forest‐plot of a random effects meta‐analysis of longitudinal studies on the association between long sleep duration and AD risk. Predictive intervals are added to the overall estimate.

The meta‐analyses of the association between long‐sleep duration and AD risk included 13 studies and showed heterogeneous results, as demonstrated by an *I* ^2^ of 77.6% (*p* < 0.001). The overall effect was 1.35 (95% CI: 1.12–1.63), and the prediction interval was 0.74–2.49, showing that the predicted effect would not be significant. (Figure 2). The funnel‐plot showed a slightly skewed distribution, but the Egger test for publication bias was not significant (*p* > 0.05) (Appendix S2, Figure S1). The meta‐regression assessing the heterogeneity and including factors such as sex, age, country of study, length of study, publication year, etc., did not reveal the reason for heterogeneity (all *p* > 0.05). Taking together, these analyses show that there might be an increased risk for AD with long sleep duration, but there was heterogeneity between the studies that remained unexplained (Table 2).

**TABLE 2:** Results of included studies.

Study Nr.	References	Risk factor (Intervention)	Result descriptive	Result measure	Potential confounders
1	Baek et al. (2021)	Insommnia	Of the 5,593,742 individuals in the non‐insomnia group, 160,660 (2.87%) during 26,305,394 person‐years, In the insomniagroup, 138,270 (4.94%) were correspondingly diagnosed with AD during 12,319,096 person‐years	Crude IR: 6.11 with no Insommnia Crude IR: 11.22 with Insommnia IRR: 1.73	Sex, Age (Inverse Correlated, The younger the more AD diagnoses per sleep disturbances) (sleep disturbance seems to have an adverse effect in a younger population)
2	Baril et al. (2021)	Sleep quantity and quality	We did not observe any significant associations between the sleep exposures and the outcomes of AD dementia	Hazard ratio (95% CI) Sleep duration: 1.09 (0.93, 1.27) *p* = 0.286 Sleep efficiency: 1.28 (0.87, 1.88) *p* = 0.215 Wake after sleep onset: 1.54 (0.85, 2.79) *p* = 0.157 Sleep latency: 0.80 (0.49, 1.31) *p* = 0.380 No. of awakenings: 1.14 (0.49, 2.63) *p* = 0.765 Arousal index: 0.73 (0.34, 1.57) *p* = 0.424 Apnea–hypopnea index: 1.12 (0.83, 1.50) *p* = 0.467	CRP measure
3	Benedict et al. (2015)	Self‐reported sleep disturbances	With sleep disturbances: 1.33‐times higher risk to develop all‐cause dementia, most pronounced for AD (+51%) AD: men with self‐reported sleep disturbance at age 70 years: 2.92 [1.76, 4.87], *p* 0.001; men with self‐reported sleep disturbance at age 50: 0.98 [0.42, 2.33], *p* 0.98.	Multivariate Cox regression analysis AD, n (% of all‐cause dementia) Sleep disturbance: 34 (47) No sleep disturbance: 85 (43) Hazard ratio: Model A 1.44 (0.97–2.14) *p* = 0.07 Model B 1.51 (1.01–2.25) *p* < 0.05 Model C 1.51 (1.01–2.25) *p* < 0.05 Model A: adjusted for age Model B: lifestyle model (age, body mass index [cont.], leisure‐time physical activity [cat.], and educational level [cat.]) Model C: adjusted for cardiometabolic factors	
4	Benito‐León et al. (2014)	Daily sleep duration 3 categories: ≤ 5 h (short sleepers) 6–8 h (reference category) ≥ 9 h (long sleepers)	The HR for dementia mortality (without non‐AD) in long sleepers remained elevated	HR for dementia mortality (non‐AD excluded): (1) long sleepers: HR = 2.09, 95% CI = 1.05–4.17, *p* = 0.04, model 1; HR = 2.13, 95% CI = 1.02–4.42, *p* = 0.04, model 2; and HR = 2.13, 95% CI = 1.02–4.42, *p* = 0.04, model 3 (2) short sleepers: HR = 0.95, 95% CI 5 0.27–3.34, *p* = 0.94, model 1; HR = 1.0, 95% CI = 0.27–3.65, *p* = 1.0, model 2; and HR = 0.99, 95% CI = 0.27–3.66, p 5 0.99, model 3	
5	Burke et al. (2016)	Sleep disturbance =nighttime behaviors (awakening during the night, rising too early in the morning, excessive naps during the day. Issues with sleep were measured utilizing NPI‐Q).	Presence of sleep disturbance was significantly associated with the eventual diagnosis of AD dementia. Presence of sleep disturbance increase the likelihood of meeting the criteria for AD dementia diagnosis during follow‐up visits among a group of cognitively intact participants at baseline.	Hazard ratio: Sleep disturbance Model 1: 2.72 (2.11–3.50)** Model 2: 3.40 (1.88–6.11)** ** *p* < 0.001 Model 1: Main effect unadjusted Model 2: Main effects adjusted for sex, age, race, maternal dementia, and paternal dementia	
6	Cavaillès et al. (2022)	sleep quality (SQ) Sleepyness during The Day (EDS) Difficulty initiating sleep (DIS) Difficulty maintaining sleep (DMS) Early morning awakening (EMA) Hypnotics use	Incident of AD Dementia was only elevated in combination with hypnotics use, anything else sleep related had no effect	Incident AD Dementia related to Hypnotics Use 1.45 [1.24–1.72] *p* < 0.0001 model 1 1.46 [1.23–1.72]*p* < 0.0001 model 2 1.32 [1.11–1.57] p0.001 model 3 Model 1: Adjusted for studycenter, sex, mobility, and presence of APOE‐E4 allele, stratified for the level of education, Model 2: Model 1 and DM, BMI and CVD Model 3: Model 2 and depressive status	
7	(Cavaillès et al. 2023)	Excessive dayime sleepiness	Excessive daytime sleepiness was associated with a higher risk of all‐cause dementia (HR 1.39, 95% CI 1.13–1.69) and DVC (HR 2.14, 95% CI 1.30–3.51) but not with AD incidence	EDS effect on AD 1.18 [0.93–1.51]	
8	Chung et al. (2025)	Sleep apnea G47.30 G47.31 G47.32 G47.38	There was a higher cumulative incidence of overall AD in the sleep apnea group than in the control group. Furthermore, after stratifying by sex, the cumulative incidence of overall AD was higher in sleep apnea in the sleep apnea group than in the control group in both men and women	AD in sleep apnea HR 1.26 [1.18–1.35] *p* < 0.001 stratified by sex: men 1.19[1.08–1.30] *p* < 0.001 women 1.38[1.24–1.53] *p* < 0.001 Adjusted HR for DAT was obtained using Cox proportional hazards regression with sleep apnea or sleep apnea subtype as a predictor after controlling for age, sex, obesity, hypertension, diabetes, hyperlipidemia, ischemic stroke, hemorrhagic stroke, coronary heart disease, physical inactivity, heavy alcohol consumption, and smoking status in each sex. Obesity was excluded as a covariate in the analysis with sleep apnea subtype as a predictor	
9	Gang and Chen (2025)	OSA conirmed through ICD‐10 codes	Over 12.6 years, 358 individuals were diagnosed with allcause dementia, including 107 with AD. Compared to those without OSA, OSA patients had significantly higher risks for all‐cause dementia	AD with OSA Model 1 1.352 [1.116–1.639] Model 2 1.291[1.062–1.569] Model 1: Adjusted for covariates including year of birth, sex, race, and education Model 2: Adjusted for competing risk of deaths and covariates including year of birth, sex, race, education, BMI, current smoking, current drinking, hypertension, diabetes, coronary heart disease, stroke, and ApoE4 carriers.	age, gender (female/male), ethnicity (white/nonwhite), educational level (high school or less/college and above), and the Townsend deprivation index for social deprivation (low, medium, high) BMI, hypertension, diabetes, stroke, and CHD
10	(Gaoet al. 2025)	daytime napping meassured with de Actical device	Cox proportional hazards models showed that a greater a.m. napping was associated with a higher risk for Alzheimer's dementia or each 1 SD increase in a.m. napping, the risk of developing Alzheimer's dementia increased by 15%	am% Incident Alzheimer's dementia Model A [HR (95% CI), p] 1.15 (1.01–1.30), Model B [HR (95% CI), p] 1.15 (1.01–1.32), Model C [HR (95% CI), p] 1.14 (1.002–1.30), Model D [HR (95% CI), p] 1.11 (0.97–1.26), Model E [HR (95% CI), p] 1.10 (0.95–1.27), pm% Incident Alzheimer's dementia Model A [HR (95% CI), p] 0.97 (0.85–1.10) Model B [HR (95% CI), p] 0.040 0.98 (0.85–1.12) Model C [HR (95% CI), p] 0.047 0.99 (0.87–1.12) Model D [HR (95% CI), p] 0.124 1.02 (0.90–1.17) Model E [HR (95% CI), p] 0.192 1.08 (0.94–1.25) m% proportion of daytime naps taken in the morning (9am–11am), HR hazard ratio, pm% proportion of daytime naps taken in the early afternoon (1pm–3pm), Model A: Covariates include age, sex, race/ethnicity, education, mean nap duration, mean nap frequency, smoking, alcohol consumption, body mass index, and physical activity level. Model B: Covariates include all Model A covariates, nighttime sleep duration, sleep fragmentation index, wake after sleep onset, interdaily stability, and intradaily variability. Model C: Covariates include all Model A covariates, depressive symptoms, thyroid disease, vascular disease risk factors, vascular disease burden, anxiety, insomnia, analgesic medication, anticonvulsant medication, beta blocker medication. Model D: Covariates include all Model A covariates and APOE ε4 carrier status. Model E: Covariates include all covariates in Models A‐D	
11	Gribsholt et al. (2025)	OSA conirmed through ICD‐10 codes	Compared with the general population cohort, OSA was associated with Alzheimer's disease	OSA compared to General Population follow up 1‐< 10y 1.18[0.96–1.45] 1–24 years 1.14[0.97–1.42]	
12	Hahn et al. (2014)	Change in sleep pattern: Self‐reported reduced duration and/or depth, measured by CPRS Rating Scale 1. Dichotomous variable: Sleep reduction > 2 h [CPRS score ≥ 1] versus sleep reduction < 2 h [CPRS score < 1] 2. Dose response analyses, 3 groups None [CPRS 0 or 0.5] Mild [CPRS 1] Moderate/severe [CPRS score ≥ 1.5] 3. Dose response analyses, continuous variable CPRS score: 0 = absence of symptoms 1 = occasional symptoms 2 = continuous symptoms 3 = “extreme” change from the person's normal behavior Additional answer choices (i.e., 0.5, 1.5, 2.5)	**1. Dichotomous variable** “Reduced sleep was associated with double the risk of AD after adjusting for age, gender, and education. The results remained after adjusting for lifestyle and vascular factors but not after adjusting for depressive symptoms.” **2. Dose response analyses, 3 groups** “Compared with no change, moderate/severe change in sleep pattern was associated with 3 times greater risk of AD (adjusted for age, education, and gender). After adjustment for depressive symptoms, the finding remained significant.” **3. Dose response analyses, continuous measure** “Compared with participants with less change in sleep pattern, greater change in sleep pattern was associated with increased risk for AD (demographics‐adjusted). This finding was also explained by depressive symptoms for AD.”	**1. Dichotomous variable:** Sleep Disturbances in Relation to AD Occurrence HR 1.98 (95% CI;1.12–3.51) *p* = 0.019 HR 2.01 (95% CI;1.12–3.61) *p* = 0.019 HR 2.09 (95% CI;1.15–3.81) *p* = 0.016 HR 1.69 (95% CI;0.91–3.14) *p* = 0.096 HR 2.02 (95% CI;1.11–3.65) *p* = 0.021 HR 1.86 (95% CI;1.00–3.47) *p* = 0.050 crude Age, gender, and education Age, gender, education, and lifestyle factors Age, gender, education, and depressive symptoms Age, gender, education, and vascular factors Age, gender, education, physical function, pain, and breathing problems **2. Dose‐response analyses:** moderate/severe change compared with no change HR: 3.11 (95% CI: 1.59–6.09) *p* = 0.001 HR: 2.77 (95% CI: 1.40–5.47) *p* = 0.003 HR: 2.91 (95% CI: 1.43–5.92) *p* = 0.003 HR: 2.19 (95% CI: 1.06–4.52) *p* = 0.034 controlled for age, education, and gender controlling for lifestyle factors controlling for vascular factors controlling for depressive symptoms **3. Dose‐response analyses:** continuous measure of change in sleep pattern HR: 1.25 (95% CI: 1.04–1.49) *p* = 0.015 HR: 1.18 (95% CI: 0.98–1.44) *p* = 0.086 demographics‐adjusted depressive symptoms adjusted	depressive symptoms
13	Huang et al. (2025)	Sleep quality	For UKB cohort, a total of 321,905 individuals free of dementia and major psychological diseases were included. During a median follow‐up of 12.3 years, 2690 incident AD events were recorded As for ADNI, a total of 1598 individuals free of dementia and major psychological diseases were included. During a mean follow‐up of 3.9 years, 293 incident AD events were recorded	UKB sleep quality good: ref. medium: 1.05 [0.97–1.14] poor: 1.28 [1.08–1.52] ADNI sleep quality good: ref. medium: 1.01 [0.76–1.132] poor: 1.62 [1.21–2.16]	
14	Kim et al. (2023)	Restless leg syndrome (RLS)	“The presence of RLS was significantly associated with an increased risk of AD.” “The incidence rate of AD was higher in the RLS group than in the control group.”	**Risk of AD between the RLS and control group:** HR 1.68 (95% CI: 1.38–2.05), *p* < 0.001* HR 1.38 (95% CI: 1.1–1.72), *p* = 0.004** * crude **adjusted for income, region, CCI score, history of sleep disorder, schizophrenia or other psychotic disorders, mood disorder, anxiety disorder, Parkinson's disease, and iron deficiency anemia **Kaplan–Meier survival curve with Gray's test** RLS group higher incidence rate of AD than control group (*p* < 0.001) **Sensitivity analyses of risk for AD in the RLS and control groups** Model 1: HR 1.23 (95% CI: 0.94–1.62), *p* = 0.133* Model 2: HR 1,36 (95% CI: 0,94–1,85), *p* = 0,055* Model 3: HR 1.37 (95% CI: 1.10–1.71), *p* = 0.006* Model 4: HR 1.76 (95% CI: 1.32–2.34), *p* < 0.001* Definition of AD by ICD‐10 codes plus anti‐dementia medications Definition of RLS by ICD‐10 code plus dopamine agonists Exclusion of patients taking antipsychotic agents Diagnosis of RLS by psychiatrists or neurologists * adjusted for income, region, CCI score, history of sleep disorder, schizophrenia or other psychotic disorders, mood disorder, anxiety disorder, Parkinson's disease, and iron deficiency anemia	RLS (other factors than sleep disturbance associated with the increased risk)
15	Kummer et al. (2019)	Cardocasvular risk factors, one of it obstructive sleep apnea (OSA)	Obstructive sleep apnea was associated with the subsequent diagnosis of AD	**Association of OSA with the Subsequent Diagnosis of AD** HR 1.25 (95% CI: 1.21–1.29)* **Censoring for Psychiatric Disorders** HR 1.14 (95% CI: 1.08–1.20)* *adjusted for age, sex, race/ethnicity, and baseline Charlson comorbidities	
16	Larsson and Wolk (2018)	Sleep duration To evaluate whether reverse causation bias may have influenced the results for sleep duration and AD risk, the analyses was repeated after exclusion of AD cases diagnosed within the first five or 10 years of follow‐up.	Long sleep duration was associated with an increased risk of dementia in the overall analysis. This association did not remain after removing cases diagnosed during the first five or 10 years of follow‐up.	**Association of sleep duration with Alzheimer's disease (AD):** (Reference: Sleep duration 7.1–9.0 h/night; 95% CI) *All participants* Sleep duration ≤ 6.0 h/night: HR 1.00 (0.92–1.09)* HR 0.99 (0.92–1.08)** Sleep duration 6.1–7.0 h/night: HR 1.02 (0.94–1.10)* HR 1.01 (0.93–1.09)** Sleep duration > 9.0 h/night: 1.56 (1.30–1.88)* 1.54 (1.27–1.85)** (1) (1) After exclusion of cases diagnosed during the first five years (HR 1.22 (0.97–1.52)) or 10 years (HR 1.03 (0.76–1.40)) of follow‐up** *Women* Sleep duration ≤ 6.0 h/night: HR 0.96 (0.86–1.07)** Sleep duration 6.1–7.0 h/night: HR 1.02 (0.91–1.14)** Sleep duration > 9.0 h/night: 1.63 (1.24–2.14)** *Men* Sleep duration ≤ 6.0 h/night: HR 1.05 (0.93–1.19)** Sleep duration 6.1–7.0 h/night: HR 1.00 (0.89–1.12)** Sleep duration > 9.0 h/night: HR 1.44 (1.11–1.86)** *Age‐ and sex‐adjusted **Age‐ and sex‐adjusted and for education, body mass index, and history of hypertension, hypercholesterolemia and diabetes, and mutually for the other lifestyle factors (except the Mediterranean diet score due to strong correlation with the DASH diet score) and sleep duration.	reverse causality (preclinical dementia leads to extended time of sleep, and not vice versa) AD diagnosis determination not clear
17	Lee et al. (2019)	SleepDisorderd breathing, CPAP or bilevel positive Airway pressure treatment	Kaplan–Meier curves of the incidence of AD based on the presence of SDB showed differences. The log‐rank test showed that the SDB group had a higher risk of AD than the non‐SDB group (log‐rank test *p* < 0.0422) Patients with an AD Diagnosis between 2002 and 2005 were excluded	HR: AD Without sleep disordered breathing: 1.00 AD with sleep disordered breathing: 1.575 (95% CI: 1,013)	
18	Li et al. (2023)	Daytime sleepiness	A longer daytime nap was associated with higher risk of developing Alzheimer's dementia with a hazard ratio (HR) of 1.20 (95% confidence interval [CI]: 1.06–1.35; *p* = 0.004; table 3) per 1 SD higher Nap duration variable. napped < 1 h/day (Figure 2C). The effect of 1 SD increase in higher nap duration was equivalent to that of being 1.6 years older at baseline (i.e., per 1 year older of age, the HR is 1.12; therefore, the HR 1.20 is corresponding to log[1.20]/log[1.12] = 1.6 years older of age; longer daytime sleepiness results in higher AD risk)	Daytime nap duration	
19	(Lin et al. 2015)	Sleep related movement disorders	12.7% of the SRMD Patients developed Dementia 3.7% of the non SRMD Patients developed dementia Patients Aged 45 to 64 had the highest risk of developing Dementia followed by patients above the age of 64 Women had higher risk of developing dementia	Crude HR *Total* All‐cause dementia 3.843 (1.918–7.698) AD 8.622 (0.781–95.163) *< 45 years* All‐cause dementia 5.127 (1.157–23.116) AD 7.255 (6.441–8.314) *45–64 years* All‐cause dementia 6.229 (1.041–37.276) AD 9.806 (8.518–10.246) *> 65 years* All‐cause dementia 4.293 (1.750–10.534) AD 6.647 (0.414–16.655) *Male* All‐cause dementia 2.303 (0.851–6.229) AD 6.124 (3.114–8.754) *Female* All‐cause dementia 6.913 (2.460–19.427) AD 8.992 (0.815–19.277)	age, sex
20	Liu et al. (2022)	Sleep duration Time in Bed (TIB) Bed Time Pittsburgh sleep quality Index Eppworth sleepiness Scale	Adjusting for multiple confounders, restricted cubic spline curves showed J‐shaped associations of sleep duration, TIB, and rise time with incident dementia, and a reverse J‐shaped association between mid‐sleep time and dementia risk Long TIB and early mid‐sleep time were significantly associated with an increased risk of dementia. There was a linear associa‐tion between bedtime and dementia risk. As a continu‐ous variable, every 1‐h advance in bedtime was associated with a 25% increased risk of dementia (95% CI 1.03–1.53). The associations of these sleep parameters with AD risk were similar to those with dementia. However, there was a reverse J‐shaped association between rise time and AD risk, and a linear association between mid‐sleep time and AD risk	*HR AD (95% CI) Model 1/Model2* *Sleep Duration* < 7 h 1.23 (0.71–2.15)/1.17 (0.67–2.05) 7–8 h 1.00 (reference)/1.00 (reference) > 8 h 1.57 (0.85–2.89)/1.54 (0.82–2.87) *Time in Bed* < 8 h 0.93 (0.5–1.73)/0.90 (0.48–1.70) 8–9 h 1.00 (reference)/1.00 (reference) > 9 h 1.47 (0.84–2.59)1.35 (0.76–2.40) *Bedtime* *>= 10p.m. 1.00 (reference)/1.00 (reference)* *10–9p.m. 1.48 (0.77–2.85)/1.55 (0.80–3.03)* *< 9p.m. 2.26 (1.15–4.46)/2.25 (1.12–4.50)* *Rise Time* < 5a.m. 1.83 (0.9–3.73)/2.04 (0.98–4.23) 5–6a.m. 1.00 (reference)/1.00 (reference) > 6a.m. 1.23 (0.62–2.44)/1.09 (0.54–2.19) *Mid‐sleep time* < 1a.m. 2.52 (1.47–4.33)/2.51 (1.45–4.34) 1–1.5a.m. 1.00 (reference)/1.00 (reference) > 1.5a.m. 0.94 (0.49–1.78)/0.92 (0.48–1.76) *Sleep quality* Good 1.00 (reference)/1.00 (reference) Poor 1.23 (0.76–2.01)/1.22 (0.75–2.00) *Sleep latency* ≤ 30min 1.00 (reference)/1.00 (reference) > 30min 1.28 (0.79–2.07)/1.31 (0.80–2.14) *Sleepefficiency* *> 85*% 1.00 (reference)/1.00 (reference) ≤ 85% 1.03 (0.64–1.68)/0.99 (0.60–1.62) Excessive *daytime sleepiness* *No* 1.00 (reference)/1.00 (reference) Yes 1.69 (0.90–3.16)/1.63 (0.86–3.09)	Adjusted for in Model 1: age, sex, education additional adjusted for in Model 2: BMI, alcohol consumption, smoking, hypertension, diabetes, dyslipidemia, coronary herat disease, stroke, and APOE genotype
21	Luojus et al. (2016)	Self‐reported sleep disturbances: Difficulty falling asleep or maintaining sleep, Sleep duration and daytime tiredness	“Men reporting frequent sleep disturbance had ~1.5‐fold increased risk for any type of incident dementia in the fully adjusted Cox analysis (model f). The association was slightly weaker with AD as an outcome, and the full model failed to reach statistical significance.” Men with frequent sleep disturbances had ~1.5 fold increased risk of AD, this result was significant regardless to all adjustments except physical activity, alcohol consumption, cumulative smoking history and in the fully adjusted model. With OSA excluded, the fully adjusted model was significant again.	Risk ratio (95% CI) for incident AD **Self‐reported sleep disturbance (never/seldom ref.)** Occasionally: 1.07 (0.80–1.42) 1.06 (0.79–1.42) 1.03 (0.77–1.37) 1.08 (0.80–1.46) 1.05 (0.78–1.40) 1.04 (0.77–1.40), * 1.07 (0.79–1.46), ** 1.26 (0.83–1.90) Often: 1.52 (1.03 to 2.23) 1.52 (1.02 to 2.25) 1.42 (0.95 to 2.12) 1.56 (1.05 to 2.33) 1.50 (1.01 to 2.23) 1.46 (0.97 to 2.20), * 1.58 (1.04–2.39), ** 1.71 (0.96–3.05) **Sleep duration (7–8 h ref.)** ≤ 6.5 h: 1.21 (0.88–1.67), ≥ 8.5 h: 0.74 (0.48–1.16) ≤ 6.5 h: 1.23 (0.89–1.70), ≥ 8.5 h: 0.75 (0.48–1.17) ≤ 6.5 h: 1.22 (0.88–1.69), ≥ 8.5 h: 0.79 (0.51–1.24) ≤ 6.5 h: 1.23 (0.89–1.70), ≥ 8.5 h: 0.75 (0.48–1.17) ≤ 6.5 h: 1.18 (0.84–1.64), ≥ 8.5 h: 0.69 (0.43–1.10) ≤ 6.5 h: 1.17 (0.84–1.64), ≥ 8.5 h: 0.73 (0.46–1.17) **Daytime tiredness (no (ref.)/yes)** 1.19 (0.89–1.60) 1.20 (0.87–1.64) 1.15 (0.84–1.59) 1.19 (0.87–1.63) 1.30 (0.93–1.81) 1.24 (0.89–1.75) Model a: adjusted for age and examination year Model b: further adjusted for Human Population Laboratory depression scale scores ≥ 5 Model c: futher adjusted for physical activity, alcohol consumption and cumulative smoking history Model d: model b further adjusted for systolic blood pressure, body mass index, low‐density and high‐density lipoprotein cholesterol, high‐sensitivity C reactive protein and cardiovascular disease history Model e: model b further adjusted for education years and living alone Model f: includes model a–e covariates * after exclusion of obstructive sleep apnoea ** after exclusion of myocardial infarction, atrial fibrillation, and heart failure	physical activity, smoking, alcohol consumption, OSA
22	(Lutsey et al. 2019)	obstructive sleep apnea (OSA) (in‐home polysomnography) short and long sleep duration (SHHS Sleep Habits Questionnaire)	“Risk of dementia or MCI due to AD tended to be higher among those with severe OSA versus a normal sleep breathing pattern after adjustment for demographics [RR Model 1: 1.66 (1.03, 2.68)], though this was attenuated in the fully adjusted models [Model 3: 1.37 (0.82, 2.30)].” “Sleep duration was not associated with risk of […], or MCI or dementia due to AD.”	Risk ratio (95% CI) for AD dementia or MCI OSA Categories (AHI) Ref.: Normal (< 5) **Mild (5–< 15)** 1.17 (0.85–1.60) 1.15 (0.83–1.58) 1.13 (0.82–1.55) **Moderate (15–< 30)** 1.32 (0.90–1.93) 1.23 (0.82–1.84) 1.25 (0.83–1.87) **Severe (> 30)** 1.66 (1.03–2.68) 1.46 (0.86–2.46) 1.37 (0.82–2.30) Sleep duration Ref.: 8 to < 9 h < 7 h: 1.25 (0.88–1.76), 7 to < 8 h: 1.01 (0.73, 1.41), > 9 h: 0.83 (0.44–1.58) < 7 h: 1.25 (0.89–1.76), 7 to < 8 h: 1.01 (0.73, 1.41), > 9 h: 0.80 (0.42–1.52) < 7 h: 1.26 (0.89–1.77), 7 to < 8 h: 1.01 (0.73, 1.41), > 9 h: 0.75 (0.40–1.43) Model 1: adjusted for age, sex, center, education level, APOE Model 2: Model 1 + body mass index, smoking status, leisure time physical activity Model 3: Model 2 + diabetes, antihypertensive medications, CRP, systolic blood pressure	MCI
23	Lysen et al. (2018)	Subjective sleep quality (home interview, Pittsburgh Sleep Quality Index PSQI)	“Poorer subjective sleep quality was not associated with […] AD.” “Several sensitivity analyses, i.e., excluding last years of the follow‐up time duration or restricting to those with best MMSE scores at baseline, did not reveal subgroups with increased risks.”	Cox regression models 320 patients developed Alzheimer's disease **Poorer subjective sleep quality and AD** HR 0.92 (95% CI 0.81–1.05)	not clear which model used to calculate HR for Alzheimer's disease not clear if subgroups analysis are only for Dementia risk or considered for Alzheimer's disease too
24	Lysen et al. (2020)	Objective estimates of sleep and 24‐h activity rhythm Measured by actigraphy	“Poor sleep as indicated by longer sleep latency, wake after sleep onset, and time in bed and lower sleep efficiency, as well as an earlier ‘lights out’ time, were associated with increased risk […], especially AD.” “Total sleep time was not associated with the risk of […] AD.” “Findings indicate that actigraphy estimated nighttime wakefulness, but not a fragmented or unstable 24‐h activity rhythm, plays a role in dementia etiology.” *For the sleep parameters, hazard ratio estimates remained mostly similar over increasing follow‐up time. The strong association of later “lights out” with lower dementia risk in the first 2 years of followup (HR 0.27, 95% CI 0.10–0.73) attenuated with increasing follow‐up time. Later L5 onset was associated with lower dementia risk in the first 2 years of follow‐up only (HR 0.23, 95% CI 0.09–0.61). Incident cases in this period all had AD. Overall, findings were similar for AD*.	Cox proportional hazards regression models 49 patients developed Alzheimer's disease *Alzheimer's disease HR 95% CI* **Sleep** Total sleep time: 0.95 (0.70–1.28) 0.92 (0.68–1.26) 0.93 (0.67–1.29) Longer sleep‐onset latency: 1.42 (1.11–1.83) 1.45 (1.11–1.89) 1.53 (1.14–2.05) Wake after sleep onset: 1.30 (1.00–1.70) 1.38 (1.05–1.81) 1.42 (1.07–1.90) Longer time in bed: 1.40 (1.01–1.95) 1.49 (1.06–2.10) 1.52 (1.07–2.15) Higher Sleep efficiency (sleep time/time in bed × 100%): 0.72 (0.54–0.94) 0.66 (0.50–0.87) 0.63 (0.46–0.86) **Bed times** Later Time ‘lights out’ 0.55 (0.40–0.76) 0.53 (0.37–0.74) Time getting up 0.81 (0.58–1.13) 0.79 (0.56–1.13) **24‐h rhythm** Intradaily variability 1.04 (0.78–1.40) 1.05 (0.78–1.41) Interdaily stability 0.90 (0.67–1.21) 0.87 (0.65–1.17) L5 onset = least active consecutive 5 h of the day 0.85 (0.65–1.12) 0.88 (0.67–1.16) adjusted for age and sex additionally adjusted for educational level, employment status, physical activity, alcohol consumption, bodymassindex, smoking status, history of cardiovascular disease, presence of hypertension, presence of diabetes mellitus additionally adjusted for 24‐h activity rhythm parameters	same population as Lysen 2018, embedded in the Rotterdam Study
25	Ohara et al. (2018)	Daily sleep duration (self‐administered questionnaire)	“The age‐ and sex‐adjusted incidence of all‐cause dementia, AD, and VaD were significantly higher in subjects with daily sleep durations of less than 5.0, 8.0 to 9.9, and 10.0 or more hours than in those with 5.0–6.9 h.” “After adjustment for [… ], (model 2) the risk of developing all‐cause dementia was significantly higher in subjects with daily sleep duration of less than 5.0, 8.0 to 9.9, and 10.0 or more hours than in those with 5.0–6.9 h. These associations were unchanged after adjusting for use of hypnotics […]. With regard to subtypes of dementia, similar associations were observed for AD and VaD.”	197 subjects with AD **Daily Sleep Duration and Risk of AD (HR, 95% CI)** Daily sleep duration **< 5 h** 2.38 (1.01–5.61)¹/2.16 (0.85–5.48)* 2.18 (0.92–5.14)^2^/2.01 (0.79–5.11)* 2.18 (0.92–5.15)^2^/2.01 (0.79–5.11)* **5.0–6.9 h:** reference **7.0–7.9 h** 1.07 (0.67–1.62)/0.98 (0.63–1.52)* 1.09 (0.72–1.67)/1.02 (0.66–1.59)* 1.10 (0.72–1.69)/1.03 (0.66–1.60)* 8.0–9.9h 1.55 (1.07–2.26)¹ /1.29 (0.86–1.92)* 1.51 (1.03–2.21)¹/1.23 (0.82–1.84)* 1.54 (1.05–2.26)¹/1.23 (0.82–1.85)* ≥ 10.0 h 2.35 (1.38–3.99)¹/1.67 (0.90–3.07)* 2.36 (1.38–4.03)¹/1.70 (0.92–3.15)*^2^ 2.43 (1.42–4.16)¹/1.72 (0.93–3.19)*^2^ Model 1: adjusted for age and sex Model 2: Model 1 + adjusted for education level, systolic blood pressure, antihypertensive agent, diabetes mellitus, hypercholesterolemia, body mass index, electrocardiographic abnormalities, history of stroke, smoking habits, alcohol intake, regular exercise Model 3: Model 2+ adjusted for use of hypnotics ^1^ *p* < 0.05 ^2^ *p* < 0.10 * after exclusion of subjects who developed dementia during the initial 3 years of follow‐up	possibility that individuals with prodromal dementia were included
26	Schneider et al. (2022)		As sleep duration increased, risk of death due to AD also increased. In particular, 9+ hours of sleep was associated with 50% increase in AD mortality risk when compared to the reference group (HR (95% CI) 1.50 (1.17, 1.92)). There was suggestive evidence of an association between short sleep duration and lower AD mortality, but the associations were not statistically significant Removing deaths within the first five years had little impact on the results	HR for death due to AD Model 1Model2/Model3/Model3excl. Sleep Duration: < 5 h 0.77 (0.52–1.12)/0.74 (0.50–1.09)/0.73 (0.49–1.08)/0.79 (0.52–1.18) 5–6 h 0.94 (0.83–1.06)/0.92 (0.82–1.05)/0.91 (0.80–1.03)/0.94 (0.83–1.08) 7–8 h reference > 9 h 1.51 (1.18–1.92)/1.50 (1.17–1.92)/1.47 (1.15–1.88)/1.43 (1.10–1.87) Nap Duration: None: reference < 1 h: 0.98 (0.87–1.10)/0.96 (0.85–1.08)/0.96 (0.85–1.09)/0.95 (0.84–1.08) > 1 h: 1.36 (1.14–1.62)/1.29 (1.08–1.55)/1.28 (1.07–1.55)/1.25 (1.03–1.53) Model 1: Adjusted for age and sex Model 2 (main model): Adjusted for covariates in Model 1 and race, education, marital status, smoking status, physical activity, alcohol use, heart disease, stroke, diabetes, cancer, sitting time, and television time Model 3: Adjusted for covariates in Model 2 and nap Model 3 excl: Model 3 after excluding deaths that occurred within 5 years after baseline	
27	Selbæk‐Tungevåg et al. (2023)	Probable insomnia disorder (PID) according to DSM‐5 criteria Insomnia symptoms difficulties initiating sleep (DIS) difficulties maintaining sleep (DMS) early morning awakenings (EMA)	“PID was not associated with AD in any of the models, DMS was associated with a lower risk of AD in the fully adjusted model, EMA was associated with a higher risk of AD only in the unadjusted model, and DIS was not associated with AD in any of the models.”	**Odds ratios (95% CI) of having AD at follow‐up by PID, DIS, DMS or EMA at baseline** **PID and AD** 1.14 (0.79–1.63) *p* = 0.495 1.21 (0.82–1.79) *p* = 0.331 1.07 (0.71–1.60) *p* = 0.755 **DIS and AD** 1.06 (0.83–1.37) *p* = 0.631 0.97 (0.74–1.26) *p* = 0.809 0.87 (0.66–1.15) *p* = 0.331 **DMS and AD** 0.82 (0.66–1.02) *p* = 0.076 0.79 (0.63–1.00) *p* = 0.051 **0.73 (0.57–0.93) *p* = 0.012** **EMA and AD** **1.31 (1.04–1.66) *p* = 0.022** 1.17 (0.91–1.50) *p* = 0.212 R 0.98 (0.75–1.27) *p* = 0.858 Model 1: unadjusted Model 2: adjusted for age and sex Model 3: additional adjusted for marital status, level of education, BMI, hypertension, history of stroke, history of diabetes, history of myocardial infarction, history of heart failure, history of COPD, sleep apnea, physical activity, smoking, alcohol consumption, HADS‐D score, HADS‐A score and ApoE	
28	Sun et al. (2025)	Sleep duration (hours) 4 groups: 0–6 h 6–9 h 9–11 h ≥ 11 h	“Both long‐term sleep (≥ 9) and short‐term sleep (< 6) increased the risk of all‐cause dementia, AD and VD.” “When the analyses were stratified by sex, we observed similar findings.” “Doseresponse analyses using restricted cubic spline also showed an approximate ‘J’ shape between sleep duration and […] AD […], that is, both shorter and longer sleep duration increased the risk […] compared to 8 h of sleep duration.” “There was a synergistic effect between mood index and sleep duration, that is, people with a low mood index and short sleep duration (< 6 h) had the highest risk of dementia.”	Sleep Duration (h) and AD (HR, 95% CI)* (Ref.: 6–9 h of sleep) **All** (0–6 h) 1.20 (1.01, 1.42), *p* = 0.042 (9–11 h) 1.22 (1.07, 1.40), *p* = 0.0031 (≥ 11 h) 2.11 (1.36, 3.29), *p* = 0.0009 **Male** (0–6 h) 1.05 (0.79, 1.38), *p* = 0.7495 (9–11 h) 1.07 (0.89, 1.30), *p* = 0.4685 (≥ 11 h) 2.43 (1.37, 4.30), *p* = 0.0024 **Female** (0–6 h) 1.31 (1.05, 1.64), *p* = 0.0173 (9–11 h) 1.39 (1.15, 1.68), *p* = 0.0006 (≥ 11 h) 1.74 (0.87, 3.50), *p* = 0.1204 *adjusted for age at baseline, race/ethnicity, years of education, income level, smoking status, physical activity level, availability of leisure activities, BMI, hypertension status, type 2 diabetes status, APOE4 allele status.	
29	Tsai et al. (2020)	OSA	among patients with OSA, 32 patients with AD were identified in a mean (SD) follow‐up period of 3.9 (2.6) years, whereas among patients without OSA, 48 patients with AD were identified in a mean (SD) observation period of 4.4 (2.9) years.	**Multivariate Cox Proportional Hazards Model for the Association of AD With OSA** adjusted OR OSA Full model 2.17 (1.3–3.51) 0.002 OSA main Model 2.80 (1.79–4.38) <.001	arrhythmia,26 coronary artery disease (CAD),26, 27 diabetes mellitus (DM),26 hypertension,26 stroke,28 heart failure,27 hyperlipidemia,29 head injury,29 depression,30 and anxiety,31
30	Virta et al. (2013)	sleep duration (hours) 4 groups: 0–6 h 6–9 h 9–11 h ≥ 11 h		Odds ratios (OR) and 95% CI of Alzheimer disease associated with sleep characteristics Unadjusted Model/Model1/Model2 *Sleep length* < 7 h 1.53 (0.99–2.36)/1.59 (0.99–2.55)/1.50 (0.92–2.45) 7–8 h 1/1/1/(reference) > 8 h 1.69 (1.08–2.63)/1.74 (1.06–2.87)/1.75 (1.07–2.88) *Insufficient sleep* No 1/1/1 (reference) Yes 0.89 (0.56–1.42)/1.15 (0.70–1.91)/1.09 (0.64–1.84) *Sleep Quality* Well 1/1/1 (reference) rather good 1.21 (0.84–1.75)/0.91 (0.61–1.36)/0.91 (0.60–1.40) Poor/rather poor 1.71 (1.02–2.89)/1.32 (0.75–2.33)/1.14 (0.61–2.15) *Snoring* Never/sometimes 1/1/1 (reference) Often/almost always 0.94 (0.62–1.45)/1.17 (0.72–1.90)/1.17 (0.71–1.91) *Use of hypnotics* Under 60 days/year 1/1/1 (reference) at least 60 days/year 1.93 (0.94–3.97)/1.67 (0.80–3.50)/1.39 (0.61–3.61) Model 1 adjusted for age, sex, education, ApoE status, and Follow‐up Model 2 adjusted for life satisfaction, obesity, hypertension, physical inactivity, heavy drinking, and binge drinking in addition to the factors adjusted for in Model 1.	
31	Wang et al. (2025)	OSAS	About 920 of 193600 individuals with OSAS and 2219 of 536701 matched individuals without OSAS developed Alzheimer's disease during the follow‐up period, corresponding to crude incidence rates of 0.81 and 0.83 per 1000 person‐years, respectively. After adjustment, the difference was not statistically significant between individuals with prevalent or incident OSAS and those without OSAS	OSAS on AD unadjusted: 0.95 [0.88–1.02] adjusted A: 1.07 [0.99–1.16] adjusted B: 1.09[1.01–1.18] Model A: Adjusted HR estimated from a competing Cox proportional hazards model adjusting for the following covariates: index year, age, sex, BMI categories, ethnicity, Townsend, Smoking status, drinking status, type 1 diabetes, type 2 diabetes, hypertension, atrial fibrillation, heart failure, myocardial infraction, stroke or transient ischaemic attack, Peripheral vascular disease, hypothyroidism, hyperthyroidism, chronic kidney disease, chronic liver disease, rheumatoid arthritis, osteoarthritis, gout, multiple sclerosis, depression, Anxiety, deafness/hearing loss, age‐related macular degeneration, cataracts, glaucoma, B12 deficiency, metformin, other glucose‐lowering drugs, lipid‐lowering drugs, angiotensinreceptor blocker/angiotensin‐converting enzyme inhibitors, other antihypertensive drugs, anticoagulants, antiplatelets, hemoglobin A1c (HbA1c) categories, estimated Glomerular filtration rate (eGFR) categories, systolic blood pressure categories, diastolic blood pressure categories, total cholesterol categories, high‐density lipoproteincholesterol categories, low‐density lipoprotein‐cholesterol categories, triglycerides categories and data source Model B: Sensitivity analysis limiting the study participants to individuals with an incident diagnosis of OSAS and their corresponding matched individuals without OSAS. BMI, body mass index; CPAP, continuous positive airway pressure; NA, not applicable; OSAS, obstructive sleep apnoea syndrome	
32	Westwood et al. (2017)	Sleep duration (self‐reported) < 6 h (short) 6–9 h (reference) > 9 h (long)	“A statistical trend […] suggested a possible association between transitioning to sleeping > 9 h and an increased risk of AD.”	Sleep duration and risk of incident AD (HR, 95% CI), no MCI at baseline* **Sleep duration at baseline** (≤ 9 h ref.) > 9 h: 1.63 (0.82–3.23) *p* = 0.17 **Former sleep duration (13 years before baseline)** (≤ 9 h ref.) > 9 h: 1.01 (0.47–2.19) *p* = 0.98 **Change in sleep between former and baseline time points** (ref. = ≤ 9/> 9 h at former and baseline time points) Change from > 9 h to ≤ 9 h: 1.39 (0.64–3.02) *p* = 0.40 Change from ≤ 9 h to > 9 h: 2.52 (1.26–5.04) *p* = 0.009 *adjusted for age, sex, education, APOE e4 allele status, homocysteine	
33	Wong et al. (2023)	Sleep duration (self‐reported) < 7 h (short) 7–8 h (normal) ref. > 8 h (long) and daytime naping	During the first 5 years of follow‐up, short versus normal sleep dura‐tion was associated with a lower dementia risk, but this changed to a positive association during 15+ years of follow‐up, with short sleep duration associated with a marginally higher dementia risk	Short versus normal sleep duration: AD: 1.04 (0.97–1.11) Long versus normal sleep duration AD: 0.96 (0.88–1.05)	education, exercise, smoking, BMI, Alcohol consumption, hormonal Therapy due to menopause, marital status, work, depression, anxiety, hypertesion, self percepted health
34	Xiong et al. (2024)	Sleep disturbnces assesed through the following questions: Difficulty falling asleep, waking up several times during the night, and Waking up with tiredness	In the analysis of the association between sleep and AD, 6930 participants were included, with 116 (1.7%) incident AD cases diagnosed by a physician	Difficulty in falling asleep (1) 0.64 [0.39–1.12] (2) 0.65 [0.38–1.11] Frequent awakenings (1) 0.66 [0.45–0.97] (2) 0.67 [0.45–0.99] Awakening tired (1) 0.85 [0.51–1.42] (2) 0.81 [0.48–1.40] Sleep duration Ideal sleep (7–8 h) (1) ref (2) ref Short sleep (< 7 h) (1) 0.71 [0.46–1.09] (2) 0.74 [0.48–1.15] Long sleep (> 8 h) (1) 2.06 [1.19–3.55] (2) 1.84 [1.04–3.24] Overall sleep quality (1) 0.75 [0.46–1.24] (2) 0.74 [0.44–1.24] Model 1: Each sleep item in the model was adjusted for age, sex, education, wealth, marriage, BMI, smoking, drinking, physical activity level, depressive symptoms, and chronic disease. Model 2: The same covariates as in model 1, but adjusting for the competing risk of death with multivariate Fine and Gray Sub distribution Hazards. In all models, the association between each sleep item and dementia was entered separately	
35	Yaffe et al. (2015)	Sleep disturbance (ICD‐9) any sleep disturbance (780.5) apnea (780.51, 780.53, 780.57) insomnia (780.52)	“Sleep disturbance in general and the subcategory diagnoses of sleep apnea and insomnia were similarly associated with Alzheimer disease (HRs from 1.19 to 1.26).”	**Association Between Sleep Diagnoses and Alzheimer's Disease, HR (95% CI)** Sleep disturbance: 1.19 (1.06–1.34) *p* = 0.003 Sleep apnea: 1.20 (1.01–1.42) *p* = 0.04 Insomnia: 1.26 (1.08–1.47) *p* = 0.003 Adjusted for baseline diabetes, hypertension, myocardial infarction, cerebrovascular disease, obesity, depression, income tertile, education	
36	Zhao et al. (2025)	Sleep duration	The restricted cubic spline (RCS) curves demonstrated nonlinear associations between weekday sleep duration and the hazard ratio of all‐cause dementia, AD, and VaD. Optimal sleep durations associated with the lowest dementia hazard ratio were 8.33 h (HR, 0.72; 95% CI 0.58–0.89) for AD	WRS Duration sleep deprived group AD 0.84 [0.701–1.006] p0.058	
37	Allwright et al. (2023)	Sleepness/insomnia	The highest‐ranking protective factor was “Sleeplessness/Insomnia”. Explanation: U‐shape	Cases: 2090 controls: 154,119: *χ* ^2^: never/rarely: 0.0392 (***); sometimes: 0.0331 (ns); usually: 0.0298 (***)	Genetic variants APOE4
38	Atayde et al. (2022)	Nighttime behavior (NTB) as unusual sleep patterns	NTB and post mortem AD diagnosis: Our findings indicate that NPI‐Q assessed nighttime behaviors – a surrogate for sleep disturbances – at onset predict faster cognitive deterioration in participants eventually found to suffer from AD	NTB‐: −2.45 points/y cognitive decline; NTB+: −3.30 points/y; *p* = 0.016	Genetic variants APOE4; cardiovascular risk; sleep medication not assessed
39	Dunietz et al. (2021)	PAP treatment and adherence of OSA	“PAP treatment and adherence are independently associated with lower odds of incident AD diagnoses in older adults” “potentially protective role for PAP therapy and continuous treatment with PAP on AD risk in older adults with OSA”	AD total: 1057 OSA treatment: Unadjusted OR 0.60 (95% CI;0.52 to 0.69) Adjusted* OR 0.78 (95% CI;0.69 to 0.89) OSA treatment adherence: Unadjusted 0.62 (95% CI;0.53 to 0.72) Adjusted* OR 0.65 (95% CI;0.56 to 0.76) *Adjusted for sex, age, race/ethnicity, stroke, hypertension, cardiovascular disease, and depression	Short follow‐up retrospective study design

**FIGURE 3:**
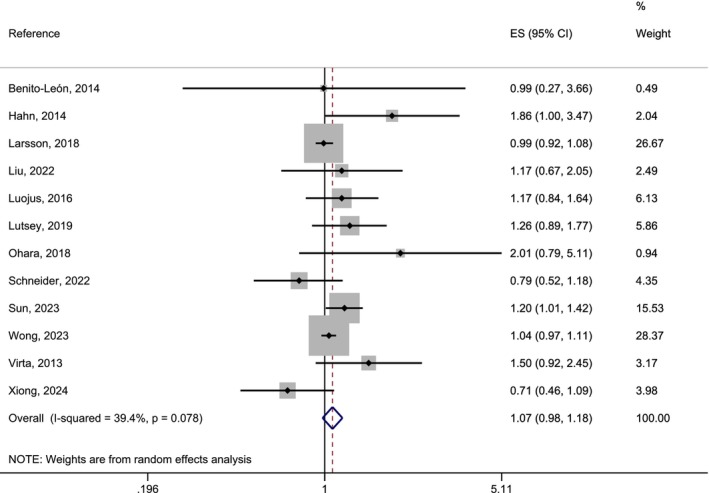
Forest‐plot of a random effects meta‐analysis of longitudinal studies on the association between short sleep duration and AD risk.

The association of short‐sleep duration and AD risk included 12 studies and showed a non‐significant result of 1.07 (95% CI: 0.98–1.18). The *I* ^2^ statistics did not reveal significant heterogeneity (Figure 3). The funnel‐plot showed a skewed distribution (Appendix 2, Figure S2), but the Egger test for publication bias was not significant (*p* > 0.05). We therefore cannot confirm a significantly increased risk for AD with short sleep duration.

**FIGURE 4:**
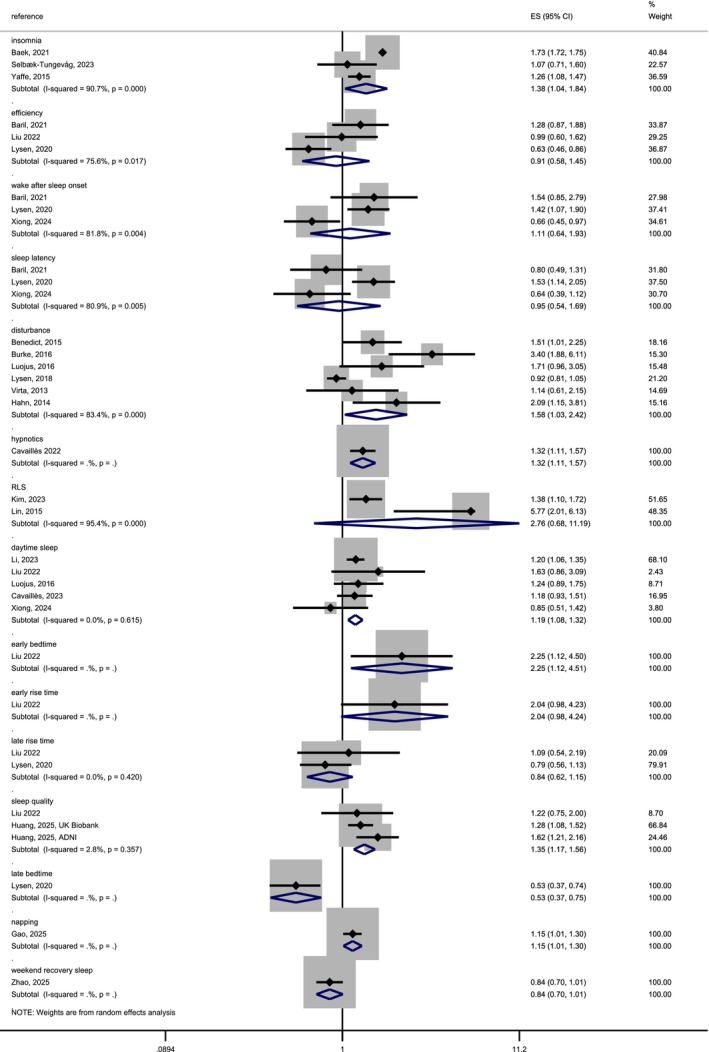
Forest‐plot of a random effects meta‐analysis of longitudinal studies on the association between sleep quality and AD risk.

The meta‐analysis of sleep quality was performed stratified by type of quality measure (15 subcategories) and included 19 studies in total. As mentioned in the method section, the sleep quality subcategories consisted of diverse phenotypes with potentially distinct mechanisms. Six out of 15 subcategories showed heterogeneity; in 4 subcategories there was only one study. For the subcategory “Daytime sleepiness” the HR of five studies was 1.19 (95% CI 1.08–1.32) with no heterogeneity. For the category “late rise time” the HR of two studies was 0.84 (95% CI 0.62–1.15) with no heterogeneity. For the subcategory “Sleep quality” the HR of three studies was 1.35 (95% CI 1.17–1.56) with no heterogeneity. For all results see Figure 4. The funnel‐plot showed skewed distributions in the subcategories (Appendix S2, Figure S3). We did not perform an overall Egger test for publication bias and meta‐regressions assessing the heterogeneity because of the low number of studies within the subcategories. Taken together, these analyses show that there might be an increased risk for AD with bad sleep quality, as well as protective factors increasing sleep quality, but to a different extent depending on the subcategories. In some subcategories there is heterogeneity between the studies that remained unexplained.

**FIGURE 5:**
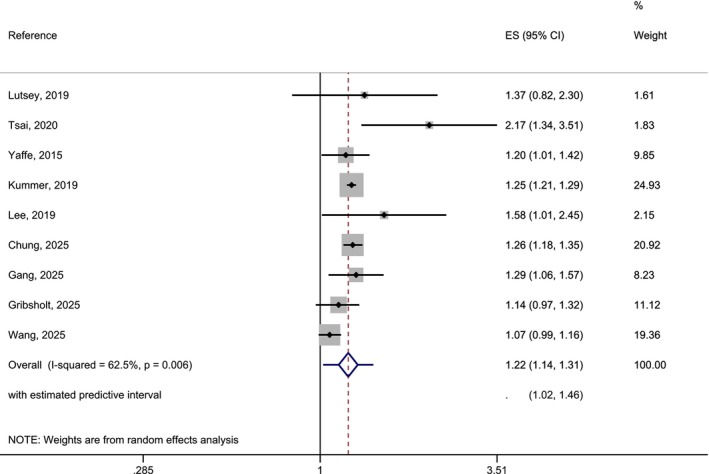
Forest‐plot of a random effects meta‐analysis of longitudinal studies on the association between OSA and AD risk. Predictive intervals are added to the overall estimate.

The association of obstructive sleep apnea (OSA) and AD risk showed a significant result of 1.22 (95% CI: 1.14–1.31). The *I* ^2^ statistics showed heterogeneity (*p* < 0.05) (Figure 5). The prediction intervals were 1.02–1.46. The funnel‐plot showed a slightly skewed distribution, but the Egger test for publication bias was not significant (*p* > 0.05) (Appendix S2, Figure S3).

## 4 Discussion

In this systematic review we investigated the effect of sleep characteristics, as well as the effect of obstructive sleep apnea, on the risk of incident AD. The 39 included studies showed good quality (NOS 6–8), and 35 of them were included in the meta‐analysis. Overall, our review on the association between sleep duration and the risk of AD was in line with other studies reporting a U−/or J‐shape association (Li et al. 2024; Fan et al. 2019; Wang et al. 2024) showing an increased risk for AD in people with short (mostly indicated as < 7 h) or long (mostly indicated as > 9 h) sleep duration. Interesting is recent research that shows that especially a change of sleep duration from short sleep to long sleep increases the risk of AD (Westwood et al. 2017) with different hypothesized pathophysiological mechanisms. For instance, short sleep duration is assumed to be associated with higher amyloid beta accumulation (Lucey et al. 2018; Spira et al. 2013). Furthermore, daytime napping duration seems to follow a U‐shaped pattern concerning the risk for AD as well, with some differences between ethnicities (Liao et al. 2025). Likewise, sleep regularity seems to be associated with AD in a U‐shaped pattern, with a balanced sleep regularity, neither too rigid nor too irregular, showing the lowest association to AD risk. This intermediate group also showed the highest levels of brain‐derived neurotrophic factor (BDNF), a biomarker for cognitive function (Cao et al. 2025).

From an evolutionary perspective our results are in line with the hypothesis (Nesse et al. 2017) of a tradeoff between the advantages of a sleep shorter than in other apes, offering more opportunities for daily activities (e.g., socializing, vigilance), and the risk of AD due to short sleep. A daily sleep time of 7–8 h seems to reflect the best trade off, marking the sleep time with the smallest risk for AD. Studies suggested that due to the reduction of sleep duration compared to other apes, and the increase in cortical neuron density, humans needed to evolve more efficient sleep (Nesse et al. 2017; Barton and Capellini 2016). The accumulation of amyloid‐beta in short sleep, but not in 7–8 h of sleep, may indicate a maximum of increased effectiveness of the removal function from toxic brain metabolites of the glymphatic system (Xie et al. 2013), especially from amyloid‐beta. Furthermore, due to sleep efficiency, extra‐long sleep of more than 9 h may not give a further advantage and takes time from daytime activities, so that an average sleep time of 7–8 h seems to be the optimal tradeoff.

However, some pharmacological studies disagree with the hypothesis of amyloid‐beta accumulation in AD, showing that even with removal of the amyloid‐beta plaques from the brain, no change of AD symptoms can be observed (Digma et al. 2024). However, these studies included populations already suffering from AD. Furthermore, previous systematic reviews found an effect of sleep disturbance only on amyloid beta but not on tau levels (Chen et al. 2025). In non‐industrialized human populations, such as the Tsimane in Bolivia, brain atrophy and dementia are rarer than in industrialized societies (Irimia et al. 2021; Gatz et al. 2023). Likewise, in other primates, brain aging shows different characteristics than in humans, with less severe outcomes (Toledano et al. 2012; Isidro 2024). The pathophysiological meaning of amyloid beta and tau accumulation in AD therefore needs further investigation.

The increased risk of AD associated with long sleep duration is assumed to have a different, but still unclear pathophysiological mechanism (Schneider et al. 2022). Recent research indicates a circadian dysfunction with disturbance of melatonin and hypocretin levels (Westwood et al. 2017; Sun et al. 2025). Additionally, since long sleep duration can be a symptom of comorbidities as well, for example, depression, there could be a confounding effect (long sleep as a symptom of depression, and depression increases the AD risk). Depression can also increase the risk for AD independently from long sleep (Young et al. 2025). Interestingly, Westwood et al. found that particularly a change of sleep habit from short sleep to a longer sleep duration increases the risk of AD (Westwood et al. 2017).

Our results on the association of long sleep duration and AD showed high heterogeneity. We could not explain the heterogeneity by population, age, or time of diagnosis. Particularly, sex did not seem to influence the association between sleep duration and AD. We therefore suspect further relevant factors responsible for heterogeneity, such as undiagnosed comorbidities, differences in population structure, or differences in sleep ecology. Heterogeneity may therefore be informative in an evolutionary mismatch framework, delivering hints under which conditions an association shows to be relevant and under which conditions it does not. To our knowledge, except to Westwood et al., no study investigated the effect of change in sleep on the risk for AD, rather than the length of sleep per se; hence this could explain a part of heterogeneity. Furthermore, Zhao et al. demonstrated the influence of socioeconomic status and lifestyle on AD risk (Zhao et al. 2024). The interplay between such potential confounders and the association between sleep duration and AD risk in different populations needs further exploration.

In terms of qualitative sleep factors, hypnotics use, daytime sleepiness, bad overall sleep quality, and early bedtime showed an increased risk for AD in our analysis, while late bedtime showed a protective effect. All in all, our results are therefore in line with the importance of restorative sleep for brain health. However, our meta‐analysis showed high heterogeneity in many subcategories, such as insomnia, sleep efficiency, wake after sleep onset, sleep latency, sleep disturbance, and RLS. Again, we could not explain the heterogeneity with age, sex, source of population, or method of diagnosis. One reason might be the heterogeneity in the definitions of sleep disturbances, sleep time boundaries, or way of collecting data (actigraphy versus self‐report). Furthermore, some authors studied potential effect modifiers in the association between sleep quality and AD. Baril et al. found that systemic inflammation moderates the effect of sleep characteristics on AD risk, with higher inflammation levels being associated with a higher AD risk (Baril et al. 2021). Similarly, Huang et al. described an association between biomarkers for neuroinflammation and AD risk, especially in carriers of the APOE E4 allele (Huang et al. 2025). Furthermore, Ferini‐Strambi pointed to a high heterogeneity in sleep assessment methodology as a possible source for heterogeneity in the association between sleep characteristics and AD risk (Ferini‐Strambi 2024). Further studies are therefore needed to unravel the confounding or moderating factors influencing the association between sleep quality and AD risk.

From an evolutionary perspective, modern sleep habits have markedly changed in the last two centuries in Westernized cultures. High use of electronic devices (Silvani et al. 2022; Li et al. 2020), high stress (Mao et al. 2023) and lower physical activity impair today's sleep quality (Alnawwar et al. 2023). This contrasts with sleep habits in non‐industrialized societies in Tanzania, Namibia, and Bolivia, where the most relevant factor governing sleep patterns seems to be the nocturnal ambient air temperature (Yetish et al. 2015). A study on positive selection of the circadian rhythm genes PER1, PER2, PER3 in different populations worldwide showed that living conditions, climate, and other external factors directly influenced the genetic structure of populations depending on geographic locations. Several of these selected variants are involved in biological processes that are associated with major modern diseases, including AD (Mishina et al. 2024).

A further fact that points toward an interaction between lifestyle factors and genetic risk variants in the risk of AD is the observation that in non‐industrialized societies the carriers of ApoE4 do not show an increased risk for AD but may even profit from higher fertility (Trumble et al. 2023) and better cognitive performance at mid age in the case of heterozygotes (Trumble et al. 2017). In contrast to this, in industrialized societies with a modern lifestyle, ApoE4 is associated with a higher risk for AD (Bufill et al. 2013). The change in sleep habits in industrialized environments and associated altered sleep quality could therefore contribute to today's high risk of developing AD.

In terms of OSA, our results showed an increased risk for AD in the presence of OSA. This result is congruent with other systematic reviews (Bubu et al. 2020; Tian et al. 2024; Kang et al. 2022). From an evolutionary perspective, OSA and its associated risk for AD seem to be linked to evolutionary changes in human anatomy and therefore represent a mechanical problem at first sight. In fact, the evolution of advanced speech and upright walking were key human adaptations that introduced anatomical changes such as the lowering of the larynx and the reduction of the maxillomandibular complex, which together resulted in a narrower and more collapsible airway. These changes could have rendered humans more susceptible to developing OSA (Torre et al. 2019). Modern conditions such as obesity (Jehan et al. 2017) or elevated alcohol consumption (Simou et al. 2018) aggravate this risk.

Overall, the results of this systematic review and meta‐analysis are consistent with previous systematic reviews indicating increased risk of AD due to a shift in sleep duration or disturbances of sleep. However, the high heterogeneity in several results indicates potential confounding factors beyond the ones that we could test for and indicates that the associations between sleep and AD may be context‐dependent. We therefore interpret AD as a multifactorial disease with probable multiple gene–environmental interactions. Our findings are therefore congruent with the evolutionary mismatch hypothesis. Mechanisms such as antagonistic pleiotropy and positive natural selection were possibly involved in maintaining AD risk alleles in the gene pool.

### 4.1 Strengths and Limitations

The strengths of this literature review include its in‐depth and comprehensive search of the available English literature. We didn't set a time limit to have an extensive overview of all available work. Besides searching for associations between sleep characteristics and AD risk, we interpreted the results in the light of evolutionary hypotheses, which represents a novel and promising approach. In fact, evolutionary explanations can deliver a deeper insight into the reasons why some anatomical or pathophysiological predispositions exist in humans and might indicate novel approaches toward prevention or treatment of diseases (Williams and Nesse 1991). Especially in the fields of neurology and psychiatry, advances in evolutionary medicine delivered promising insights (Nesse 2023). In the field of AD research, several evolutionary hypotheses were proposed (Fox 2018; Nesse et al. 2017; Isidro 2024; August et al. 2025; Finch and Sapolsky 1999), and we included most of them in the discussion of our results.

The limitations of our study include the fact that we only looked at literature in English. Furthermore, AD is often associated with sleep disruption, a fact that could lead to reversed causality (Chen et al. 2024). As sleeping problems can show as the first symptom of AD, as well as a risk factor for it, it is difficult to differentiate between the two possibilities (Ju et al. 2014). Most studies that we analyzed approached this problem by defining any signs of cognitive impairment as exclusion criteria at baseline. One study explicitly tested for a potential reverse causality bias by running a sensitivity analysis excluding AD cases diagnosed within the first five or ten years of follow‐up (Larsson and Wolk 2018). However, for other studies we could not exclude the possibility that prodromal cases were included at baseline (Ohara et al. 2018). As this aspect is crucial for our discussion, future cohort studies must explicitly address this issue.

Furthermore, because of the unresolved heterogeneity in our meta‐analyses all interpretations must be carried out with caution; while there are some overall significant associations (e.g., between long sleep duration and AD), the high heterogeneity and correspondingly wider prediction intervals indicate that these associations are not constant across study designs or populations. Possible untested confounders might include sleep medication and comorbidities that influence sleep or cognition (e.g., depression, alcohol consumption, malnutrition, cardiometabolic diseases, etc.). Additionally, there might be heterogeneity among studies in the definitions of sleep disturbances, sleep time boundaries, or in data collection methods (actigraphy versus self‐report). In general, heterogeneity could be informative from an evolutionary perspective, as it could indicate under which conditions an association seems to be relevant and under which conditions it does not. Finally, as it is difficult to differentiate AD from other dementia forms in the living patient, some misclassification in the primary literature cannot be excluded.

## 5 Conclusion and Outlook

In conclusion, this systematic review showed a possible association between sleep characteristics and the risk for AD, but with high heterogeneity in several outcomes. The fact that we could not reveal the reasons for the heterogeneity of our results might indicate that AD is influenced by a plethora of different risk factors other than sleep, and that there are potentially unknown confounders. Therefore, more studies are needed to better understand the development of AD risk, as for instance the interplay between different risk factors affecting AD (Uleman et al. 2024). This information could be used to unravel the complex interplay between AD risk factors and disease outcomes, and might mirror evolutionary influences that are not present anymore in Western societies.

Overall, we confirmed that long sleep as well as short sleep and bad sleep quality seemed to increase the risk for AD, with the lowest AD risk being an undisturbed sleep of 7–8 h. The fact that this sleep duration is distinctly shorter from the sleep duration of other apes hints toward an association of sleep duration and brain development in human evolution. Furthermore, the fact that non‐Westernized societies seem not to show the same association between the main genetic risk variant APOE E4 and AD hints toward gene‐environment interactions. These results support the concept of an evolutionary mismatch with modern environments as potential explanations for the association of different sleep characteristics and AD risk in Westernized societies and evolutionary trade‐offs might have modulated this association. Furthermore, mechanisms such as antagonistic pleiotropy and positive natural selection were possibly involved in maintaining AD risk alleles in the gene pool. In conclusion, more studies are needed on the association of changing sleep characteristics and AD risk (Westwood et al. 2017)., especially to further clarify under which conditions associations between sleep characteristics and AD show in Western societies, mirroring evolutionary processes.

Supporting Information: For this paper Supporting Information is available in the supplementary section.

## Funding

This study was financed by the Institute of Evolutionary Medicine.

## Ethics Statement

The authors have nothing to report.

## Conflicts of Interest

The authors declare no conflicts of interest.

## Supporting information


**Appendix S1:** full search strategy.
**Appendix S2:** Funnel plots of the meta‐analyses.
**Figure S1:** Funnel plot with pseudo 95% confidence intervals of the random effects meta‐analysis of longitudinal studies on the association between long sleep duration and AD. X‐axis with estimates on the log scale. The red line is the fitted line corresponding to the regression test for funnel‐plot asymmetry (Egger test).
**Figure S2:** Funnel plot with pseudo 95% confidence intervals of the random effects meta‐analysis of longitudinal studies on the association between short sleep duration and AD. X‐axis with estimates on the log scale. The red line is the fitted line corresponding to the regression test for funnel‐plot asymmetry (Egger test).
**Figure S3:** Funnel plot with pseudo 95% confidence intervals of the random effects meta‐analysis of longitudinal studies on the association between OSA and AD. X‐axis with estimates on the log scale. The red line is the fitted line corresponding to the regression test for funnel‐plot asymmetry (Egger test).

## Data Availability

Data sharing not applicable to this article as no datasets were generated or analysed during the current study.
