# Supplementary material for: Systematic Review on the Association Between Sleep and the Risk of Alzheimer’s Disease: An Evolutionary Perspective
Source: Evol Appl. 2026 Mar 23;19(3):e70223. doi: 10.1111/eva.70223 (PMC13093591; doi:10.1111/eva.70223)

**Systematic review on the association between sleep and the risk of Alzheimer’s disease: an evolutionary perspective**

Spirig, SE^1^, Frei, S^1^, Jaeggi, A^1^, Bender, N^1^

^1^Institute of Evolutionary Medicine, University of Zurich, Winterthurerstrasse 190, 8057 Zurich, Switzerland

**Appendix 1: full search strategy**

**Pubmed**

(((sleep[Title/Abstract]) OR (Sleep Wake Disorders[MeSH Terms]) OR (sleep quality[Title/Abstract]) OR (sleep duration[Title/Abstract])) AND ((Alzheimer Disease[MeSH Terms) OR (alzheimer*[Text Word]) OR (alzheimer's disease[Title/Abstract]) OR ((dementia AND Alzheimer[Title/Abstract])) OR (dementia[MeSH Terms]) OR (abp, alzheimer s[MeSH Terms]) OR (abp, alzheimer's[MeSH Terms]) OR (alzheimer abp[MeSH Terms]) OR (alzheimer beta protein[MeSH Terms]) OR (alzheimer disease[MeSH Terms]) OR (alzheimer dementia, presenile[MeSH Terms]))) NOT (letter[Publication Type]) NOT (comment[Publication Type]) NOT (review[Publication Type]) NOT (systematic review[Publication Type]) NOT (editorial[Publication Type]) NOT (meta-analysis[Publication Type]) NOT (rat[MeSH Terms]) NOT (mouse[MeSH Terms]) NOT (drosophila[MeSH Terms])

**Embase**

('sleep disorder'/exp OR 'sleep disorder' OR 'sleep time'/exp OR 'sleep time') AND ('alzheimer disease'/exp OR 'alzheimer disease' OR (('dementia'/exp OR dementia) AND ('alzheimer disease'/exp OR 'alzheimer disease'))) NOT ('letter'/exp OR letter OR 'review'/exp OR review OR 'editorial'/exp OR editorial OR 'rat'/exp OR rat OR 'mouse'/exp OR mouse OR 'drosophila'/exp OR drosophila)

**Appendix 2: Funnel plots of the meta-analyses**

**Figure S1**


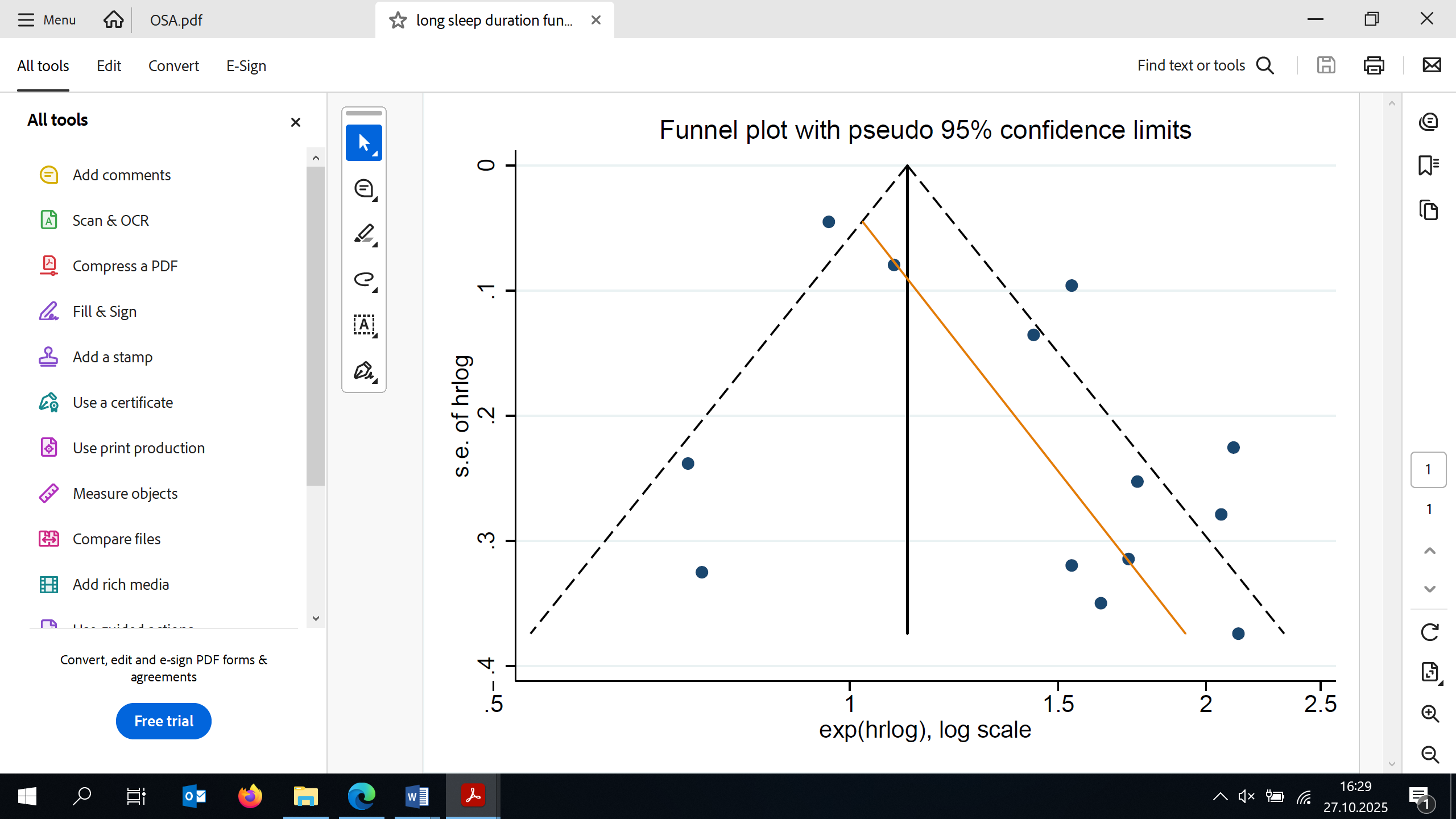


**Figure S1:** Funnel plot with pseudo 95% confidence intervals of the random effects meta-analysis of longitudinal studies on the association between long sleep duration and AD. X-axis with estimates on the log scale. The red line is the fitted line corresponding to the regression test for funnel-plot asymmetry (Egger test).

**Figure S2**


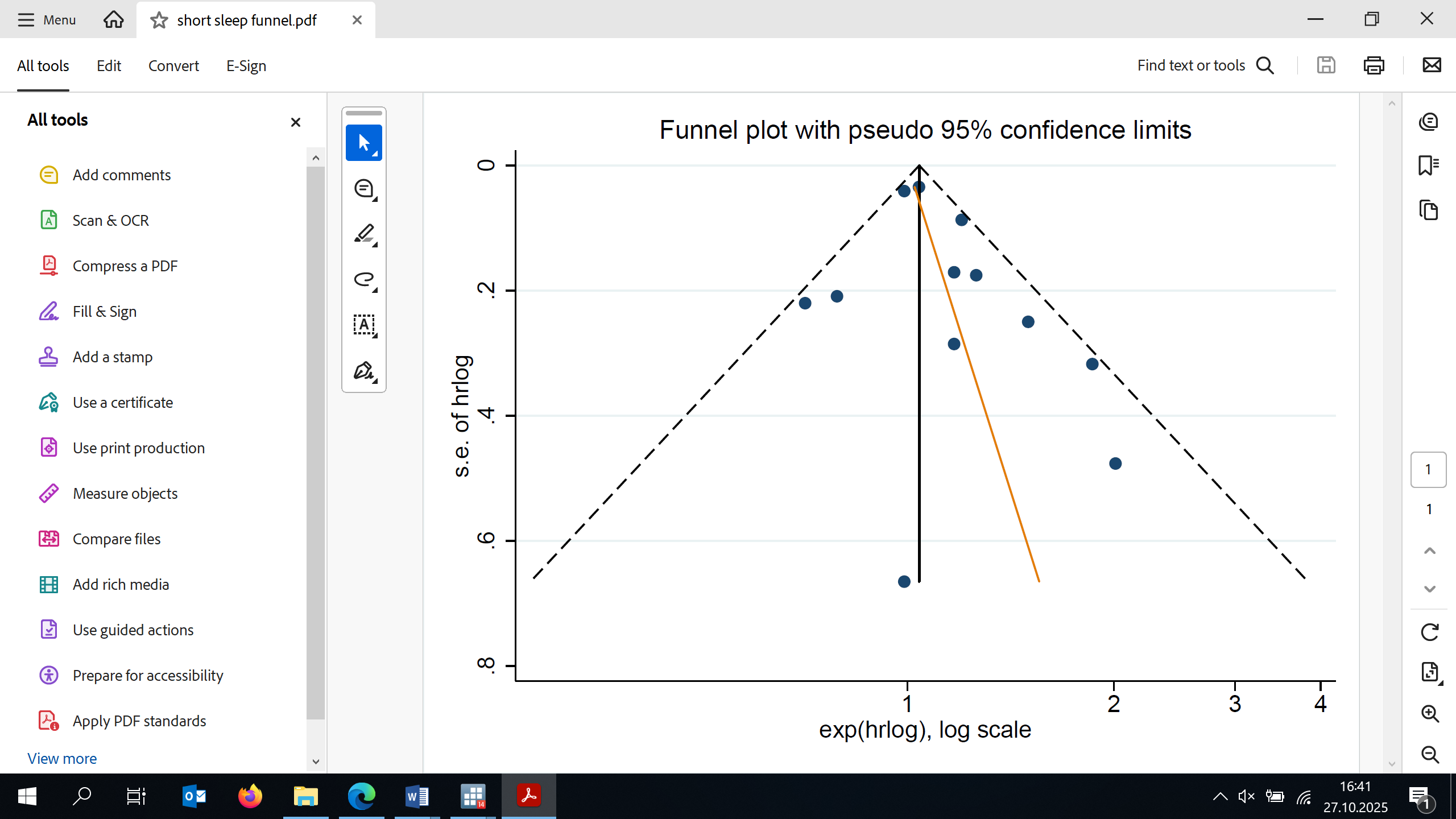


**Figure S2:** Funnel plot with pseudo 95% confidence intervals of the random effects meta-analysis of longitudinal studies on the association between short sleep duration and AD. X-axis with estimates on the log scale. The red line is the fitted line corresponding to the regression test for funnel-plot asymmetry (Egger test).

**Figure S3**

**Figure S3:** Funnel plot with pseudo 95% confidence intervals of the random effects meta-analysis of longitudinal studies on the association between OSA and AD. X-axis with estimates on the log scale. The red line is the fitted line corresponding to the regression test for funnel-plot asymmetry (Egger test).


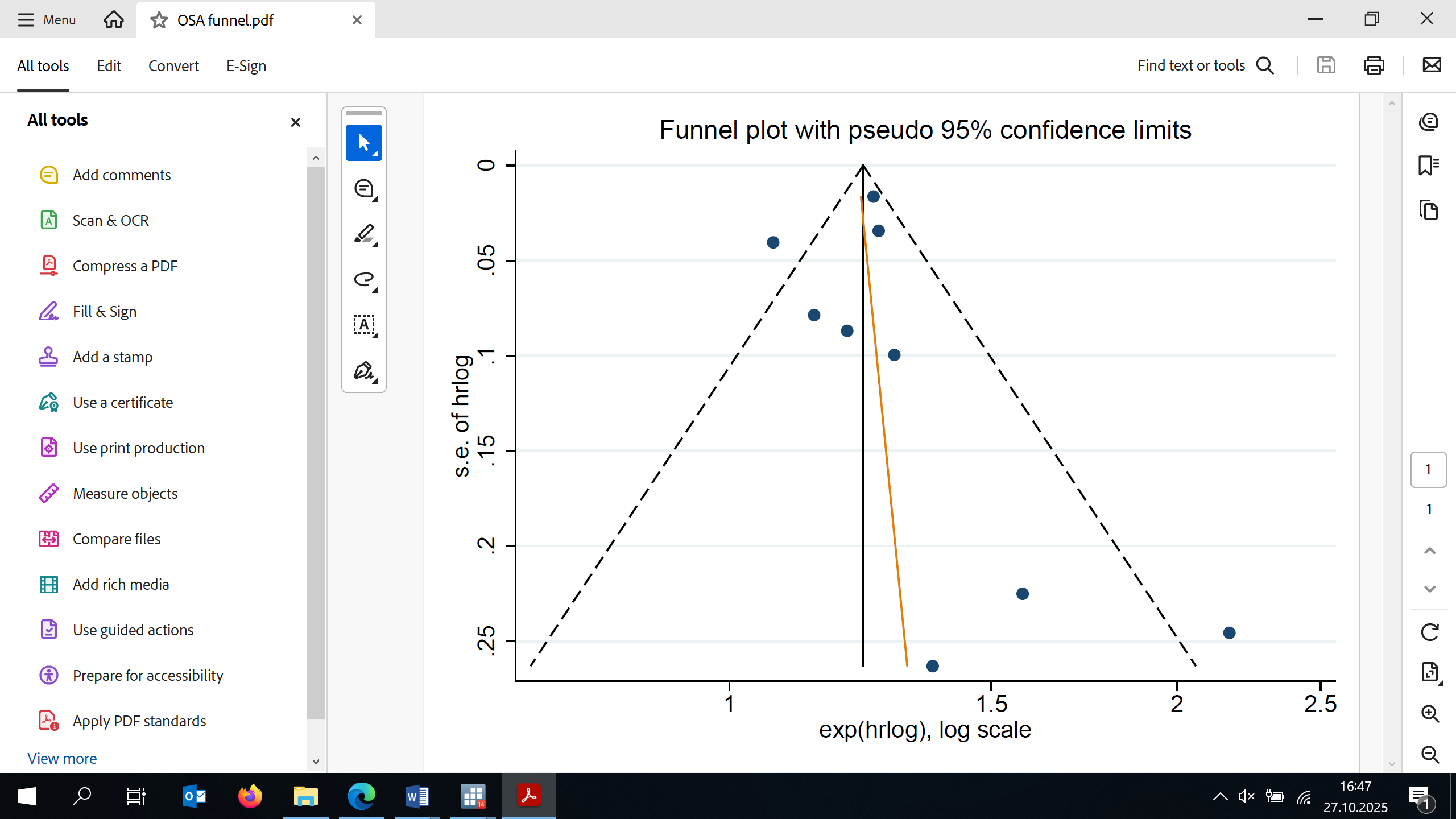

Supplement: Supplementary file 1 — Appendix S1: full search strategy. Appendix S2: Funnel plots of the meta‐analyses. Figure S1: Funnel plot with pseudo 95% confidence intervals of the random effects meta‐analysis of longitudinal studies on the association between long sleep duration and AD. X‐axis with estimates on the log scale. The red line is the fitted line corresponding to the regression test for funnel‐plot asymmetry (Egger test). Figure S2: Funnel plot with pseudo 95% confidence intervals of the random effects meta‐analysis of longitudinal studies on the association between short sleep duration and AD. X‐axis with estimates on the log scale. The red line is the fitted line corresponding to the regression test for funnel‐plot asymmetry (Egger test). Figure S3: Funnel plot with pseudo 95% confidence intervals of the random effects meta‐analysis of longitudinal studies on the association between OSA and AD. X‐axis with estimates on the log scale. The red line is the fitted line corresponding to the regression test for funnel‐plot asymmetry (Egger test). [file EVA-19-e70223-s001.docx]
